# Aficamten is a small-molecule cardiac myosin inhibitor designed to treat hypertrophic cardiomyopathy

**DOI:** 10.1038/s44161-024-00505-0

**Published:** 2024-07-23

**Authors:** James J. Hartman, Darren T. Hwee, Julien Robert-Paganin, Chihyuan Chuang, Eva R. Chin, Samantha Edell, Ken H. Lee, Roshni Madhvani, Preeti Paliwal, Julien Pernier, Saswata Sankar Sarkar, Julia Schaletzky, Kristine Schauer, Khanha D. Taheri, Jingying Wang, Eddie Wehri, Yangsong Wu, Anne Houdusse, Bradley P. Morgan, Fady I. Malik

**Affiliations:** 1 Research and Non-Clinical Development, Cytokinetics, South San Francisco, CA USA; 2 Structural Motility, Institut Curie, Université Paris Sciences et Lettres, Sorbonne Université, CNRS UMR144, Paris, France; 3 Tumor Cell Dynamics Unit, Inserm U1279 Gustave Roussy Institute, Université Paris-Saclay, Villejuif, France

**Keywords:** Drug discovery, Cardiology

## Abstract

Hypertrophic cardiomyopathy (HCM) is an inherited disease of the sarcomere resulting in excessive cardiac contractility. The first-in-class cardiac myosin inhibitor, mavacamten, improves symptoms in obstructive HCM. Here we present aficamten, a selective small-molecule inhibitor of cardiac myosin that diminishes ATPase activity by strongly slowing phosphate release, stabilizing a weak actin-binding state. Binding to an allosteric site on the myosin catalytic domain distinct from mavacamten, aficamten prevents the conformational changes necessary to enter the strongly actin-bound force-generating state. In doing so, aficamten reduces the number of functional myosin heads driving sarcomere shortening. The crystal structure of aficamten bound to cardiac myosin in the pre-powerstroke state provides a basis for understanding its selectivity over smooth and fast skeletal muscle. Furthermore, in cardiac myocytes and in mice bearing the hypertrophic R403Q cardiac myosin mutation, aficamten reduces cardiac contractility. Our findings suggest aficamten holds promise as a therapy for HCM.

## Abstract (web summary)

Hartman et al. use mouse models of cardiac function to show that aficamten decreases the availability of myosin heads for contraction during systole, attenuating a primary driver of hypertrophic cardiomyopathy pathophysiology.

## Main

HCM is the most common monogenetic heart disease, with an estimated carrier prevalence reported to affect ~1 in 500 people^1^. Advances in genetic testing and diagnostic imaging, as well as other changes in clinical practice, have led to the suggestion that the prevalence is even higher^2^. Clinically evident disease is less common with population-based insurance claims and national health system data indicating that the prevalence of the clinical population of individuals with HCM in the United States and European Union is between 1 in 2,000 and 1 in 3,195 (refs. ^3–6^).

HCM is characterized by left ventricular (LV) hypertrophy in a non-dilated LV, often with evidence of myofibrillar disarray, fibrosis and diastolic dysfunction^7–9^. Most patients with phenotypic HCM have either a normal or increased LV ejection fraction (LVEF), and approximately 70% of patients will demonstrate LV outflow tract (LVOT) obstruction. Obstructive HCM (oHCM) is mediated by the combination of an anatomically narrowed LVOT and the interplay with the mitral valve leaflets and subvalvular apparatus^8–10^ and is associated with increased cardiac morbidity and mortality^11^. Although nonobstructive HCM is less prevalent and associated with low annual mortality rates, these patients also have high rates of adverse clinical events, including potentially higher arrhythmic risk^12^.

Autosomal dominant mutations in the sarcomere, the basic contractile unit of muscle, are the leading cause of HCM, with mutations in β-cardiac myosin (*MYH7*) and cMYBP-C (*MYBPC3*) accounting for approximately 50% of familial HCM^13,14^. A common feature uniting the diversity of genetic causes of HCM seems to be hypercontractility, manifested as increases in net sarcomere power generation in vitro^15–17^ and LV hypercontractility with diminished cardiac compliance in vivo^18^. A molecular mechanism emerging as a driver of this phenotype is an increased number of myosin heads in the thick filament that bind to actin in the thin filament during the cardiac contractile cycle^19^. In some cases, mutations may destabilize the myosin off-state, where myosin heads are typically maintained in an energy-sparing conformation positioned close to the thick filament^20–22^. In other cases, mutations can affect the behavior of thin-filament proteins and produce hypercontractility through altered calcium sensitivity, regulation or structure^23,24^.

Pharmacologic therapies for symptomatic oHCM have focused on repurposing other drugs used in cardiology for patients with HCM. These include β-adrenergic receptor blockers, non-dihydropyridine calcium channel blockers and disopyramide, all intended to either slow heart rate, reduce cardiac contractility or both. While these therapies are primarily intended to reduce symptoms, they are in some instances contraindicated or poorly tolerated due to other on- and off-target effects^25^. Implantable cardioverter defibrillators are used in patients at high risk for potentially fatal cardiac arrhythmias. When patients with obstructive HCM fail to respond or are unable to tolerate medical therapies, invasive therapies (surgical septal myectomy or alcohol septal ablation) may be recommended if available^7,13^. While surgical myectomy has demonstrated a meaningful impact on long-term prognosis^26^, patient access is limited and some postoperative patients continue to experience significant morbidity from atrial fibrillation, stroke and heart failure^27^.

The therapeutic relevance of cardiac myosin inhibition in HCM is well established. The cardiac myosin inhibitor mavacamten^28,29^ has recently received US Food and Drug Administration approval and is available for the treatment of symptomatic oHCM to improve functional capacity and symptoms. The pivotal study of mavacamten in oHCM was the EXPLORER-HCM trial. Patients treated with mavacamten in addition to standard-of-care therapy showed improved symptoms and exercise capacity in conjunction with significant reductions in LV outflow tract pressure gradients (LVOT-G)^29^. A distinctive feature of mavacamten is a long human half-life (*t*_1/2_) of approximately 7–9 days, requiring about a 6-week period to reach steady-state concentrations^30,31^. Additionally, the inhibition of mavacamten metabolism by cytochrome P450 (CYP) 2C19 inhibitors (for example, omeprazole or esomeprazole), strong CYP3A4 inhibitors or in combination with medications whose use is susceptible to CYP induction (for example oral contraceptives) complicate its use^30^.

Aficamten was engineered to optimize the pharmacology of cardiac myosin inhibition, including plasma half-life, pharmacodynamics, CYP interactions, once-daily dosing, attainment of steady-state within 2 weeks, reversibility of effects within 24–48 h and a shallow exposure–response relationship^32^. We believe that these attributes will translate into meaningful clinical advantages for patients and providers using this potential medicine. Here we provide evidence that aficamten has a distinct mechanism of action, identify its binding site via a high-resolution co-crystal structure of aficamten and cardiac myosin, as well as describe its effects on cardiac contractility.

## Results

### Aficamten potently inhibits cardiac myosin ATPase activity

**Fig. 1:**
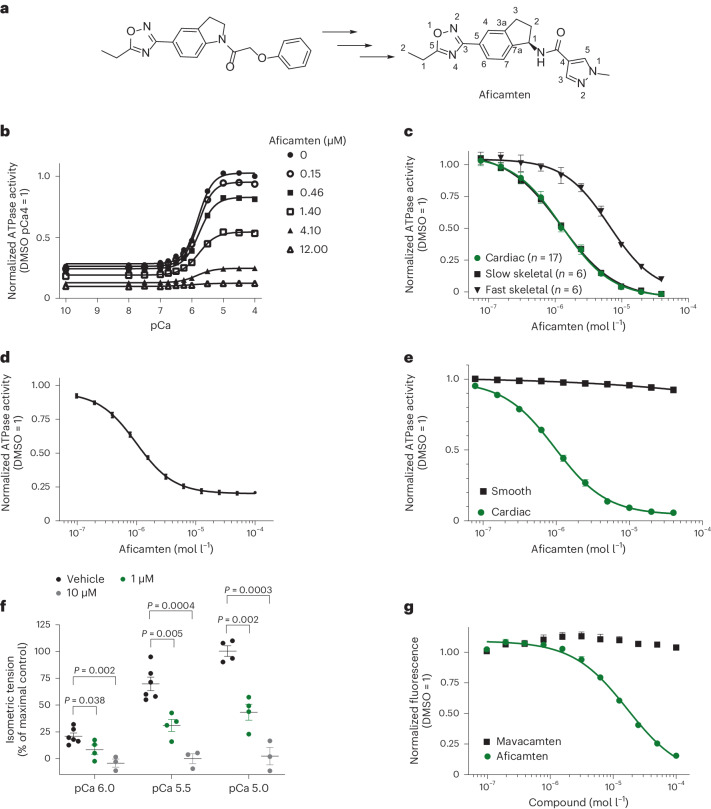
Aficamten is a selective small-molecule cardiac myosin inhibitor. **a**, The chemical structures of the initial screening hit (CK-2172010) and aficamten. **b**, Inhibition of bovine cardiac myofibril ATPase activity by aficamten. Aficamten concentrations are indicated on the graph and data. Data shown are mean ± s.d. (*n* = 4) and were fit with a four-parameter model, equation (1). pCa = −log([free Ca^2+^]) control ATPase activity = 0.26 µM s^−1^. **c**, Selective inhibition of bovine cardiac and slow skeletal versus rabbit fast skeletal myofibril ATPase activity by aficamten. Myofibrils were tested at a calcium concentration producing 75% of maximal activation (pCa_75_). Non-myosin ATPase activity was subtracted from cardiac and slow skeletal myofibril data by subtracting the ATPase activity in the presence of a saturating concentration of blebbistatin. Data shown are mean ± s.d. (cardiac *n* = 17; slow skeletal *n* = 6; fast skeletal *n* = 6) and were fit with a four-parameter model, equation (1). Average control ATPase activities: 0.043 µM s^−1^ (cardiac), 0.055 µM s^−1^ (slow skeletal), 0.17 µM s^−1^ (fast skeletal). **d**, Inhibition of bovine cardiac myosin S1 (1 µM) basal ATPase activity by aficamten. Data shown are mean values ± s.d. (*n* = 8) and were fit with a four-parameter model. Control ATPase activity = 0.029 µM s^−1^. **e**, Selective inhibition of the actin-activated (14 µM) ATPase activity of bovine cardiac myosin S1 (0.2 µM) versus chicken gizzard SMM S1 (1 µM) by aficamten. Data shown are mean values ± s.d. (*n* = 4) and were fit with a four-parameter model, equation (1). Control ATPase activities: 0.29 µM s^−1^ (cardiac), 0.47 µM s^−1^ (smooth). **f**, Inhibition of skinned rat cardiac fiber isometric force production by aficamten. Data shown are mean values ± s.e.m. (vehicle, *n* = 6; 1 µM, *n* = 4, 10 µM, *n* = 3 unique skinned cardiac fibers). *P* values shown were corrected for multiple comparison using the Holm–Sidak method (*α* = 0.05). **g**, Aficamten, but not mavacamten, reduces myosin-enhanced blebbistatin fluorescence intensity consistent with mutually exclusive binding. Data shown are mean values ± s.d. (*n* = 4 technical replicates) and were fit with a four-parameter model, equation (1). Source data

Aficamten was identified through high throughput screens for inhibitors of the cardiac sarcomere followed by iterative rounds of optimization for potency, projected human pharmacokinetics and slope of the pharmacodynamic exposure–response relationship, with the objective of maximizing safety, efficacy and ease of use for patients (Fig. 1a). Aficamten decreased the adenosine triphosphatase (ATPase) activity of detergent-washed bovine cardiac myofibrils across the full range of activating calcium concentrations in a dose-dependent fashion (Fig. 1b). Potency and selectivity were examined using myofibrils prepared from different muscle types, demonstrating that aficamten inhibited the ATPase activity of cardiac and slow skeletal myofibrils (both containing the same β-myosin heavy chain; β-MHC) with similar potencies (mean half-maximum inhibitory concentration (IC_50_), 1.26 (95% confidence interval (CI) 1.20–1.33) µM and 1.23 (95% CI 1.17–1.29) µM, respectively) and more potently than rabbit psoas fast skeletal myofibrils that contain >96% fast skeletal myosin *MYH1* (ref. ^33^) (6.52 (95% CI 5.72–7.71) µM) (Fig. 1c). Due to the high amino acid sequence conservation in vertebrates, modulators of myosins generally behave similarly across species allowing for comparisons across myofibril sources^32^.

**Extended Data Fig. 1:**
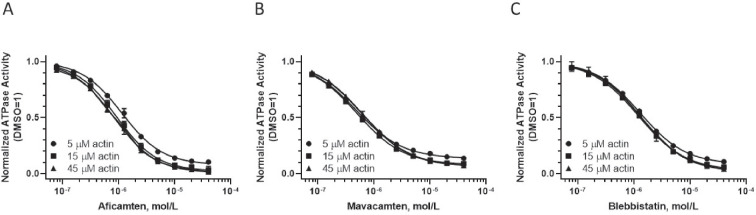
Effect of actin concentration on inhibition by myosin inhibitors. Inhibition of the actin-activated ATPase activity of bovine cardiac myosin S1 (0.2 µM) by (**A**) aficamten, (**B**) mavacamten, and (**C**) blebbistatin. Data shown are mean values ± SD (n = 4 technical replicates). Data are fit with four-parameter dose–response curves (1) with top values fixed at 1. Control ATPase activities: 0.115 µM/sec (5 µM actin), 0.258 µM/sec (15 µM actin), 0.443 µM/sec (45 µM actin). Results of curve fitting are shown in Table S1. Source data

Aficamten is a direct inhibitor of the cardiac myosin catalytic domain (subfragment-1; S1), as it reduced the steady-state ATPase activity of cardiac myosin S1 in the absence of other sarcomere proteins, including actin, troponin and tropomyosin (mean IC_50_ 1 µM, 95% CI 0.95–1.03) with similar potency as observed for cardiac myofibrils (Fig. 1d). Notably, aficamten did not inhibit the actin-activated ATPase activity of smooth muscle myosin (SMM) S1 (IC_50_ » 40 µM) as compared to cardiac myosin S1 (mean IC_50_ 0.96 µM, 95% CI 0.91–1.02) (Fig. 1e), minimizing the possibility of confounding hemodynamic effects due to vasodilation. The potency and extent of inhibition of cardiac myosin S1 actin-activated ATPase activity was insensitive to actin concentration (Extended Data Fig. 1a and Supplementary Table 1) and potencies were similar to that observed with myofibrils (where the local actin and myosin concentrations are high), reflecting intact muscle. While muscle ATPase activity and muscle force production are generally well correlated^34^, we directly tested its ability to reduce cardiac muscle force production in skinned cardiac fibers. Aficamten partially reduced isometric skinned cardiac fiber force production at 1 µM, approximately the IC_50_ in the cardiac myofibril ATPase assay (Fig. 1f). At 10 µM, a concentration that gave near-complete inhibition of cardiac myofibril ATPase activity, calcium-induced force production was minimal.

**Extended Data Fig. 2:**
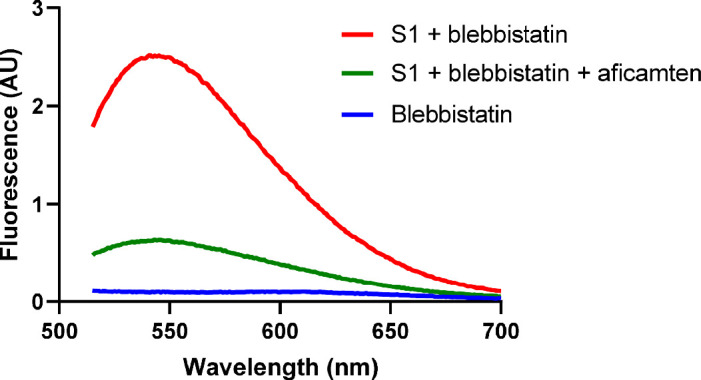
Fluorescence emission spectra of blebbistatin. Fluorescence emission intensity of (-)-blebbistatin (5 µM) increases upon binding to bovine cardiac myosin S1 (1 µM) in the ADP-VO_4_ state. This increase is attenuated in the presence of excess aficamten (50 µM). Samples were excited at 426 nm. Source data

Like aficamten, mavacamten is an allosteric small-molecule cardiac myosin inhibitor (refs. ^28,35^, Extended Data Fig. 1b and Supplementary Table 1). To better understand potential mechanistic differences between aficamten and mavacamten, we compared their ability to compete for binding to cardiac myosin with the nonselective myosin II inhibitor, blebbistatin (Extended Data Fig. 1c and Supplementary Table 1), whose binding site has been determined by X-ray crystallography^36,37^. Blebbistatin is intrinsically fluorescent and its fluorescent intensity increases upon binding to the myosin catalytic domain (Extended Data Fig. 2). In the presence of increasing concentrations of aficamten, blebbistatin fluorescence was reduced, indicating that aficamten and blebbistatin bind in a mutually exclusive manner and suggesting that they have identical or overlapping binding sites on myosin (Fig. 1g and Extended Data Fig. 2). In contrast, increasing concentrations of mavacamten had little effect on blebbistatin fluorescence in this assay, suggesting that mavacamten binds to a site on myosin distinct from blebbistatin and aficamten.

### Structural biology identifies the aficamten binding site

**Fig. 2:**
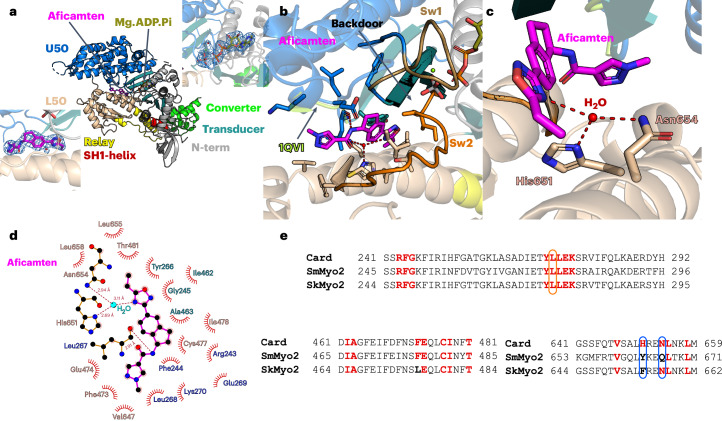
Structure of β-cardiac myosin motor domain complexed to aficamten and Mg.ADP.Vanadate in the PPS state. **a**, Overall structure with the different subdomains colored distinctly. Two insets show that both the nucleotide and the drug were rebuilt without ambiguity in the density: the upper inset displays ADP and Vanadate and the lower inset displays aficamten. In both the 2Fo-Fc electron density map is contoured at 1.0 σ of the nucleotide and the drug is shown, demonstrating that these elements have been built without ambiguity. The subdomains are colored distinctly. N-term, N-terminal subdomain. **b**, Aficamten targets the same pocket as blebbistatin. The side chains of the residues involved in the binding are shown as sticks. **c**, Zoom in on the coordination of the water involved in the binding of aficamten. The connectors switch-1 (Sw1) and switch-2 (Sw2) are colored distinctly. **d**, Scheme of the aficamten binding site drawn with LigPlot^72^. **e**, Sequence alignment of different *Homo* *sapiens* class-2 myosin heavy chains: β-cardiac myosin (Card), SMM 2 (SmMyo2), skeletal myosin 2 (SkMyo2). Positions of the residues involved in the binding of aficamten are represented in bold. If the residue is conserved, it is colored red. Non-conserved positions are colored black. Residues involved in a polar interaction are contoured in orange. Residues involved in the coordination of water are contoured in blue.

To better define the mechanism of action of aficamten and to explain its specificity for β-MHC, we co-crystallized β-MHC with aficamten and Mg.ADP.Vanadate (Fig. 2a). The structure solved at 2.33 Å resolution revealed that two β-MHC motor domain molecules were trapped in the pre-powerstroke (PPS) state in the asymmetric unit (Table 1). The electron density map unambiguously identified the position of aficamten and the nucleotide in both molecules (Fig. 2a). As suspected from competition assays (Fig. 1g), aficamten targets the same pocket as blebbistatin, located between the U50 (upper 50 kDa) and the L50 (lower 50 kDa) subdomains in close vicinity to the inorganic phosphate (Pi)-release backdoor (Fig. 2b). Little conformational change occurs upon aficamten binding, the primary difference being a new position of the Leu267 side chain. For blebbistatin and its analogs, binding to myosin requires a larger main chain rearrangement of the 267–271 loop to widen the pocket and allows formation of an essential hydrogen bond between the compound and the Leu270 carbonyl amide^37,38^. The structure suggests that the mechanism of action is similar for aficamten and blebbistatin as they occupy the same pocket in a myosin PPS state. Inhibition of ATPase activity by the compounds results from prevention of rearrangements of the myosin internal cleft, which are required to change the actin interface and thus the release of the hydrolysis products. These shifts in conformation are required upon actin binding to promote P_i_ release and to allow the motor to progress into force producing states^39,40^.

**Table 1:** Data collection and refinement statistics (molecular replacement)

	PPS-MD–AFICAMTEN
**Data collection**	
Space group	P2_1_
Cell dimensions	
*a*, *b*, *c* (Å)	69.424, 128.326, 103.530
α, β, γ (°)	90.000, 91.467, 90.000
Resolution (Å)	103.496–2.332 (2.591–2.332)
*R*_merge_ (all I+ & I−)	0.110 (1.122)
*R*_merge_ (within I+ & I−)	0.102 (1.009)
Number of observations (total)	365,861 (17,585)
Number of observations (unique)	51,951 (2,599)
*I* / σ*I*	10.4 (1.6)
Completeness (spherical) (%)	67.3 (12.5)
Completeness (ellipsoidal) (%)	93.8 (62.7)
Redundancy	7.0 (6.8)
CC_1/2_	0.998 (0.608)
Refinement	
Resolution (Å)	46.60–2.33 (2.39–2.33)
No. reflections	51,926 (254)
*R*_work_/*R*_free_	0.197/0.234
No. atoms	11,954
Protein	11,493
Ligand/ion	140
Water	321
*B*-factors	74.79
Protein	75.12
Ligand/ion	66.28
Water	63.52
r.m.s. deviations	
Bond lengths (Å)	0.016
Bond angles (°)	1.84

Values relative to the highest resolution shell are within parentheses.

r.m.s., root mean square; MD, motor domain; *R*_free_, free R-factor measured on 5% of the data excluded from refinement; *R*_work_, R-factor measuring the agreement between experimental data and the model during refinement; CC_1/2_, half-dataset correlation coefficient used for high-resolution cutoff.

**Extended Data Fig. 3:**
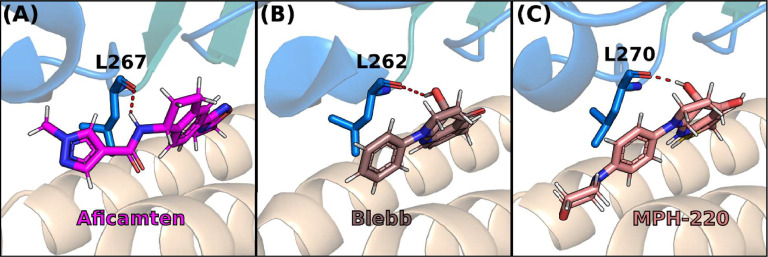
Detail of the Electrostatic Bond Involving the Carboxyl Group of a Conserved Leucine. **(A)** L267 from β-cardiac myosin interacts with the N-H from aficamten. **(B)** L262 from D. discoidum Myosin II interacts with the hydroxyl group of blebbistatin (PDB code 1YV3, ref. ^37^). **(C)** The same kind of interaction occurs between L270 from skeletal myosin II and the hydroxyl of MPH-220 (PDB code 6YSY, ref. ^38^).

A more detailed analysis of the pocket shows that most of the interactions are hydrophobic in nature with only two electrostatic interactions (Fig. 2b–d). The first electrostatic bond involves the carboxyl group of the L267 main chain that forms a hydrogen bond with the N–H moiety from the amide bond between the pyrazole and indane of aficamten (Extended Data Fig. 3a). This hydrogen bond interaction is analogous to the hydrogen bond of the angular OH moiety of blebbistatin and its analog (MPH-220) with the backbone carbonyl of L262 in *Dictyostelium* *discoideum* myosin II (Extended Data Fig. 3b)^37^ or L270 in fast skeletal muscle myosin^38^ (Extended Data Fig. 3c), respectively. The second electrostatic interaction involves a hydrogen bond network between a water molecule with the nitrogen from the imidazole of H651 and a side chain NH_2_ of N654 of β-MHC and nitrogen 4 of the oxadiazole from aficamten (Fig. 2b–d). The high resolution of the electron density map allows the visualization of the water molecule coordinated to H651 and N654 of β-MHC without ambiguity (Fig. 2a).

Despite binding to the same site as the nonselective inhibitor blebbistatin, notably aficamten is selective for cardiac β-MHC relative to smooth and fast skeletal muscle myosin. Specificity is likely achieved through the above-mentioned second electrostatic interaction involving a water-mediated hydrogen bond to residues _Car_H651 and _Car_N654 of β-MHC and nitrogen 4 of the oxadiazole from aficamten. This network of specific bonds has lost a coordinating partner in the case of fast skeletal muscle myosin (skMyo2) as _Car_H651 is replaced by the phenylalanine _Sk_F654 and this apolar side chain removes a hydrogen bond acceptor from the water hydrogen bond network for coordination with aficamten (Fig. 2d,e). For SMM, _Car_H651 is replaced by tyrosine _SmMyo2_Y663 and _Car_N654 is replaced by glutamine _SmMyo2_Q666 likely disrupting two hydrogen bonds from this network by increasing the distance (in the case of the _SmMyo2_Q666 for _Car_N654) and removing the aryl nitrogen hydrogen bond acceptor (in the case of the _SmMyo2_Y663 for _Car_H651) (Fig. 2e and Supplementary Table 2). These findings explain the selectivity of aficamten for cardiac versus fast skeletal myofibrils (5.3×) and the selectivity of aficamten for cardiac myosin S1 over SMM S1 (>40×).

### Aficamten inhibits Pi release, promoting weak actin binding

**Fig. 3:**
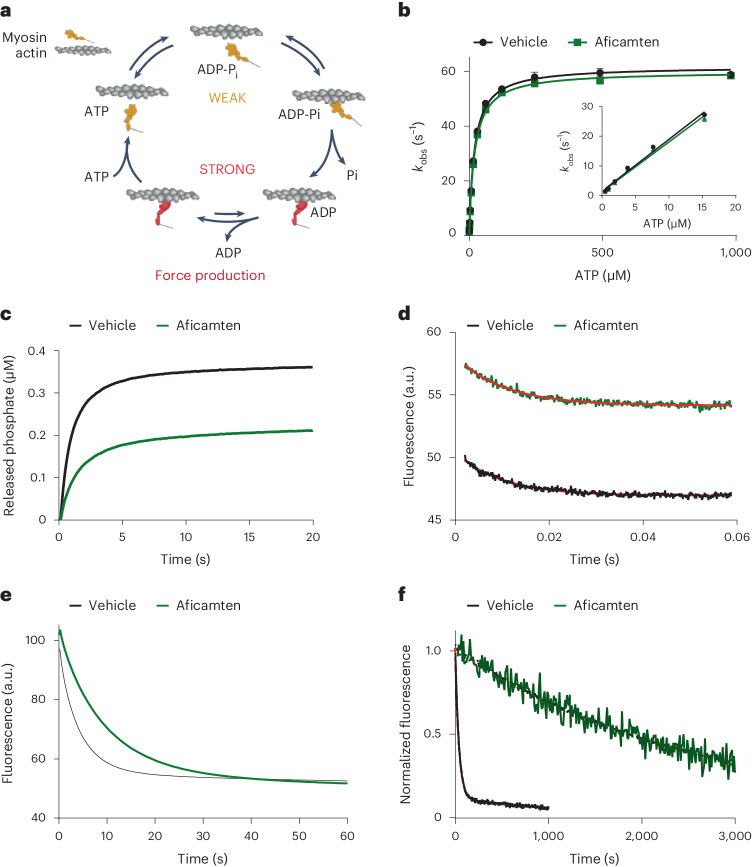
Mechanism of cardiac myosin inhibition by aficamten. **a**, Myosin mechanochemical cycle. **b**, ATP binding and hydrolysis are unaffected by aficamten. Bovine cardiac myosin S1 (1 µM final concentration) was rapidly mixed with varying concentrations of ATP while monitoring myosin intrinsic tryptophan fluorescence. Aficamten was included in all solutions at 40 µM. Three to five fluorescence transients were averaged and fit to a single exponential equation (2). Data shown are mean ± s.d. (*n* = 3 separate experiments). Vehicle, *k*_+H_ + *k*_−H_ = 62 s^−1^, ATP binding rate = 1.7 × 10^6^ M^−1^ s^−1^; aficamten, *k*_+H_ + *k*_−H_ = 60 s^−1^, ATP binding rate 1.7 × 10^6^ M^−1^ s^−1^. **c**, Actin-activated phosphate release is slowed by aficamten. Bovine cardiac myosin S1 was rapidly mixed with ATP, aged and then rapidly mixed with actin and MDCC–PBP. Final concentrations were 0.5 µM myosin, 0.25 µM ATP, 14 µM actin and 5 µM MDCC–PBP. Aficamten was included in all solutions at 40 µM. Data shown are the average of four to six transients from a representative experiment, which was well fit to a double exponential equation (3), superimposable with the data. Aggregated data are shown in Extended Data Fig. 4 and Supplementary Table 3. **d**, Actin-activated ADP release is very modestly slowed by aficamten. Bovine cardiac myosin S1 was preincubated with mant-ADP and actin followed by rapid mixing with excess ATP. Final concentrations were 1 µM myosin, 0.5 µM mant-ADP, 5 µM actin and 1 mM ATP. Aficamten was included in all solutions at 40 µM. Data shown are from a single representative reaction. The red line indicates the best fit to a single exponential equation (2). Aggregated data are shown in Extended Data Fig. 5. **e**, Basal ADP release is modestly slowed by aficamten. Bovine cardiac myosin S1 was preincubated with mant-ADP followed by rapid mixing with excess ATP. Final concentrations were 1 µM myosin, 0.5 µM mant-ADP and 1 mM ATP. Aficamten was included in all solutions at 40 µM. Data shown are the average of four to six transients from a representative experiment, which was well fit to a single exponential equation (1), superimposable with the data. Aggregated data are shown in Extended Data Fig. 6. **f**, Aficamten dramatically slows single ATP turnover. Bovine cardiac myosin S1 was preincubated with mant-ATP followed by rapid mixing with excess ATP. Final concentrations were 0.5 µM myosin, 1.0 µM mant-ATP and 1 mM ATP. Aficamten was included in all solutions at 25 µM. Data shown are from a single representative reaction. The red line indicates the best fit to a double exponential equation (3) for vehicle and a single exponential equation (2) for aficamten. Aggregated data are shown in Table 2. PBP, phosphate binding protein; MDCC, 7-diethylamino-3-((((2-maleimidyl)ethyl)amino)carbonyl) coumarin. Source data

**Extended Data Fig. 4:**
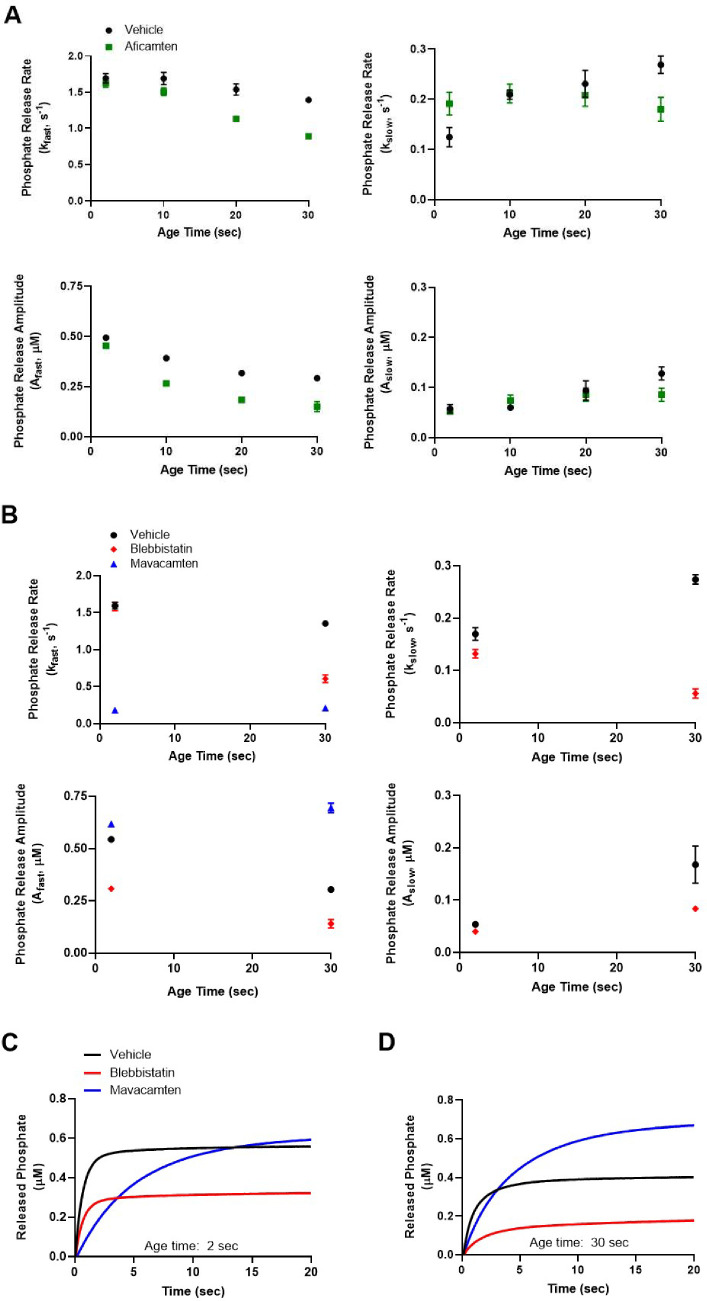
Effect of aficamten, blebbistatin, and mavacamten on actin-activated phosphate release. Bovine cardiac myosin S1 was rapidly mixed with ATP, aged, and then rapidly mixed with actin and MDCC-PBP. Final concentrations after mixing: 0.5 µM myosin, 0.25 µM ATP, 14 µM actin, 5 µM MDCC-PBP. Aficamten, blebbistatin, or mavacamten were included in all solutions at 40 µM. **(A)** Phosphate release rate and amplitude as a function of age time in the presence of aficamten. **(B)** Phosphate release rate and amplitude as a function of age time in the presence of mavacamten or blebbistatin. **(C)** Representative phosphate release reaction with 2 second age time. **(D)** Representative phosphate release reaction with 30 second age time. Data were best fit to the sum of two exponentials (3), with the exception of reactions containing mavacamten which were well fit with a single exponential (2), due to small amounts of ATP present in the actin solutions. Note that the amplitude of the fast rate of Pi release declines at longer age times under control conditions, due to loss of phosphate during the ageing period prior to mixing with actin. Data shown is the average of four to six transients from a representative experiment. (n = 5 technical replicates) Aggregated data are shown in Table S3, S4. Source data

The kinetic mechanism of aficamten was further explored by probing key steps in the myosin chemomechanical cycle (Fig. 3a) using pre-steady-state methods. Suprapharmacological concentrations were used to probe for kinetic effects on different steps of the myosin cycle at complete biochemical inhibition levels. Aficamten did not alter ATP binding or hydrolysis as measured by intrinsic tryptophan fluorescence (Fig. 3b); however, actin-activated phosphate release was dramatically slowed, as indicated by a reduction in both the rate and amplitude of phosphate release (Fig. 3c). Suppression of phosphate release by aficamten was critically dependent upon aging time with myosin following mixing with ATP (Extended Data Fig. 4 and Supplementary Table 3), suggesting modestly slow binding of aficamten to the myosin(ADP–Pi) complex. Slow binding has also been observed with blebbistatin (Extended Data Fig. 4, Supplementary Table 4 and ref. ^41^) but differs from mavacamten, which slows phosphate release similarly at both 2 and 30 s age times and produces similar phosphate release amplitudes as in the uninhibited state (Extended Data Fig. 4 and Supplementary Table 4). The modestly slow binding of aficamten complicates pre-steady-state characterization, as complete inhibition (such as what was observed in steady-state ATPase experiments) likely occurs over multiple ATPase cycles due to competition between myosin(ADP–Pi) binding aficamten versus binding actin and releasing phosphate. The complex phosphate release phenotype observed (reduction in phosphate release rate combined with reduced phosphate release amplitude) is consistent with incomplete occupancy of the aficamten binding site during the aging period of the experiment, extending into the actin-activated phosphate release phase. The modestly slow rate of aficamten binding is unlikely to be of physiological consequence, as the inhibited state can be fully populated within minutes under cycling conditions such as in the steady-state myofibril and actin-activated ATPase assays.

**Extended Data Fig. 5:**
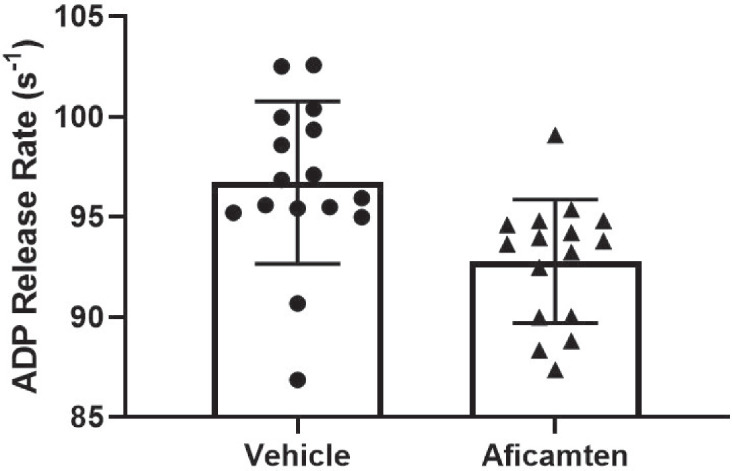
Rate of actin-activated mantADP release from bovine cardiac myosin S1 (1 µM) in the absence and presence of 40 µM aficamten. Data shown are mean +/- SD (n = 16 technical replicates from n = 4 samples). Source data

**Extended Data Fig. 6:**
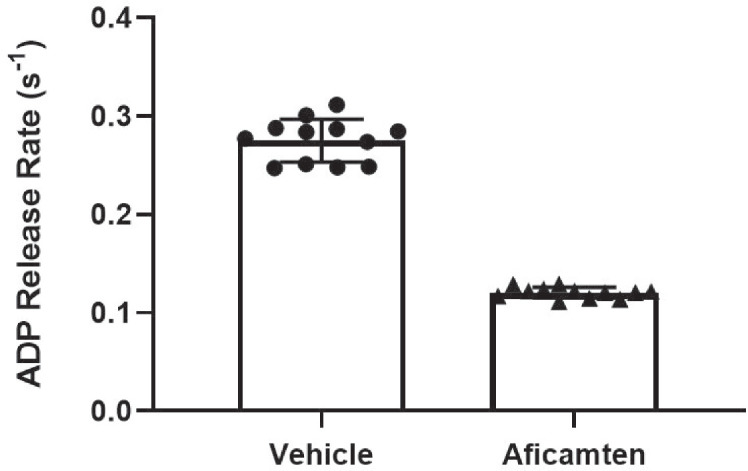
Rate of basal mantADP release from bovine cardiac myosin S1 (1 µM) in the absence and presence of 40 µM aficamten. Data shown are mean +/- SD (N = 12 technical replicates from n = 3 samples). Source data

Following phosphate release, ADP must be released from actomyosin before ATP binding and myosin detachment. Aficamten very modestly slowed ADP release from actin-bound myosin (Fig. 3d and Extended Data Fig. 5), suggesting minimal impact on the ability of myosin to exit from force-generating states. The basal rate of ADP release was slowed approximately twofold by aficamten (Fig. 3e and Extended Data Fig. 6), similar to what has been reported for both blebbistatin^41,42^ and mavacamten^35^. This result indicates aficamten can interact at least weakly with myosin in the ADP state; however, this degree of slowing should have minimal physiological impact as interaction with actin would trigger rapid ADP release and allow for unimpeded ATP binding and actin dissociation.

**Extended Data Fig. 7:**
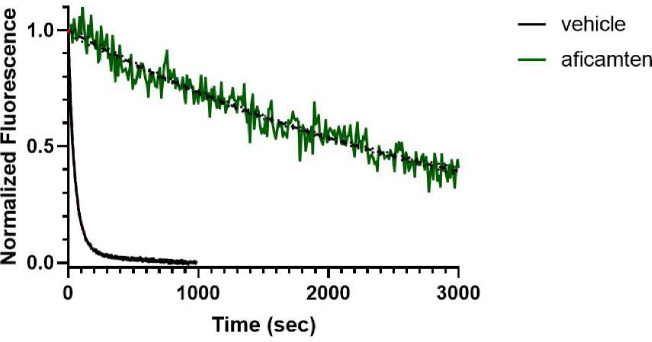
Time course of single nucleotide turnover by bovine cardiac HMM (0.5 µM) in the absence (black) and presence (green) of 25 µM aficamten. Single representative experiment is shown. The fitted lines represent the best fit to a double exponential (3) for vehicle and single exponential (2) for aficamten. Pooled data is shown in Table S5. Source data

Single ATP turnover measurements were performed using purified myosin in the absence of actin to characterize the inhibited state of myosin stabilized by aficamten. Single-headed cardiac myosin S1 has 6% slow turnover and 94% fast turnover in the absence of the compound (Fig. 3f and Table 2) as observed previously^43,44^. Addition of 25 μM aficamten had two effects on the inhibitory state of myosin–the fraction of rapidly exchanging myosin heads decreased to essentially 0% and the ATP turnover rate decreased significantly from 0.0014 s^−1^ to ~0.0004 s^−1^ (*P* < 0.0001), an even slower rate than what was reported for the super-relaxed (SRX) state by Hooijman et al.^45^. The effect of aficamten on two-headed heavy meromyosin (HMM) was similar, essentially eliminating the fraction of rapidly exchanging myosin heads and dramatically slowing the turnover rate to well below the conventional SRX rate (Extended Data Fig. 7 and Supplementary Table 5).

**Table 2:** Effect of aficamten on the single nucleotide turnover rate of cardiac myosin S1

Treatment	*N*	% Slow turnover	*k*_fast_ (s^−^^1^)	*k*_slow_ (s^−1^)
Vehicle	15	6.4 ± 4.5	0.030 ± 0.004	0.0014 ± 0.0008
25 μM aficamten	25	100^a*^	N/A	0.00043 ± 0.0002*

Data are presented as mean ± s.d. ^a^Traces for reactions containing aficamten were equally well fit by single equation (2) and double exponential equation (3). *P < 0.0001 versus vehicle calculated using a two-tailed unpaired *t*-test. N/A, not available; *k*_fast_, fast rate constant; *k*_slow_, slow rate constant.

**Extended Data Fig. 8:**
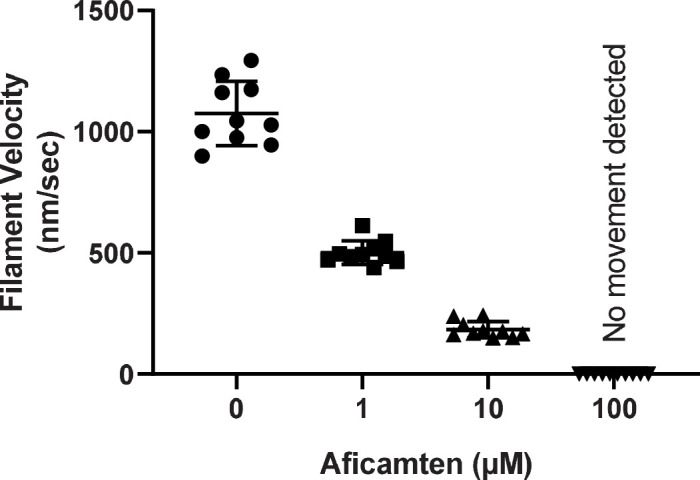
Inhibition of the rate of actin filament sliding by aficamten. Results shown are mean +/- SD (n=ten filaments from a single myosin prep) under each condition. All reactions contained 2% DMSO. Movies of actin filament sliding included as Supplementary Videos 1-4. Source data

Aficamten inhibited the in vitro motility of bovine cardiac HMM in a dose-dependent manner, reducing actin filament gliding velocity with similar potency as observed for inhibition of steady-state ATPase activity (~50% at 1 µM), while still allowing for actin attachment (Extended Data Fig. 8 and Supplementary Videos 1–4). Similar results have been previously reported for blebbistatin^36^ and mavacamten^35^.

### Aficamten reduces cardiac contractility in vitro and in vivo

**Fig. 4:**
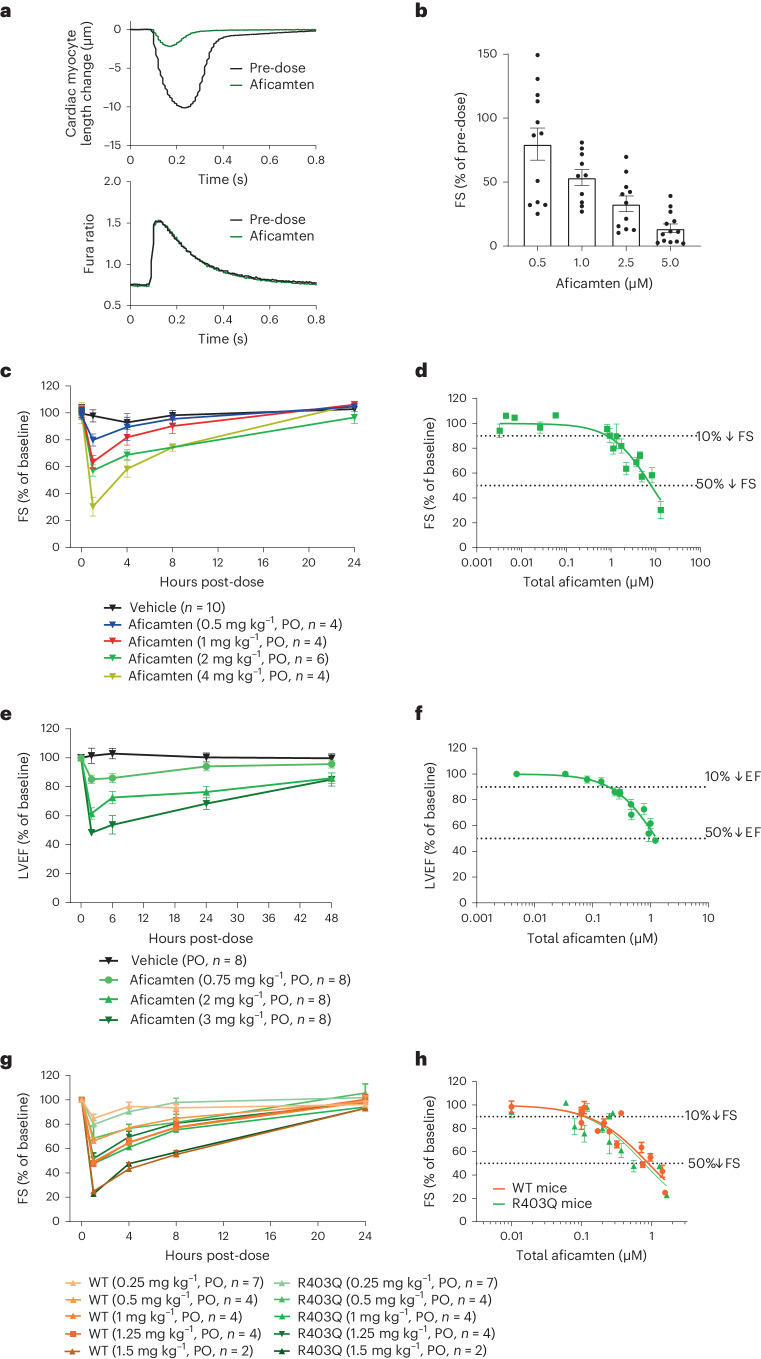
Aficamten reduces cardiac contractility in vitro and in vivo. **a**, Aficamten (10 μM) reduces myocyte contractility without altering calcium transients. Data shown are for a single representative cell. The top graph represents myocyte contractility and bottom graph represents calcium transients (intracellular free Ca^2+^ measured using the radiometric indicator Fura2, where the Fura ratio = fluorescence emission at λ_em_  510 nm when excited at λ_ex_340/380 nm, which is proportional to the free [Ca^2+^]). Aggregated data are shown in Table 3. **b**, Aficamten reduces the contractility (fractional shortening, FS) of adult rat cardiomyocytes at concentrations similar to those that inhibit myosin ATPase activity. Data shown are mean ± s.e.m. (*n* = 10–13 cardiomyocytes for each concentration prepared from *n* > 3 preparations). **c**, Aficamten reduces FS of healthy rats. Echocardiographic measurements of FS before (baseline, BL) and 1, 4, 8 and 24 h after single oral doses (PO) of aficamten in healthy rats. FS is shown as a percent of baseline values. Data are shown as mean ± s.e.m. (vehicle, *n* = 10; 2 mg kg^−1^; aficamten, *n* = 4; 0.5, 1 and 4 mg kg^−1^ aficamten, *n* = 4 per group). **d**, FS reduction in normal rats as a function of total plasma aficamten concentration. Rat FS concentration response curve was generated by plotting the average plasma concentration and the average FS response at each time point in the four sets of animals dosed at 0.5, 1, 2 and 4 mg kg^−1^ (*n* = 16 total data points). Values shown are mean ± s.e.m. **e**, Echocardiographic measurements of ejection fraction before (BL) and 2, 6, 24 and 48 h after single oral doses of aficamten in beagle dogs. LVEF is shown as a percent of baseline values. Data are shown as mean ± s.e.m. (*n* = 8 per dose group). **f**, Dog LVEF concentration response curve was generated by plotting the average total plasma concentration and the average LVEF response at each time point in dogs dosed at 0.75, 2 and 3 mg kg^−1^ (*n* = 8 per dose). Values shown are mean ± s.e.m. **g**, Echocardiographic measurements of FS before (BL) and 1, 4, 8 and 24 h after single oral doses of aficamten in WT and R403Q mice. FS is shown as a percent of baseline values. Data are shown as mean ± s.e.m. (0.25 mg kg^−1^, *n* = 7 per group; 0.5, 1 and 1.25 mg kg^−1^, *n* = 4 per group; 1.5 mg kg^−1^, *n* = 2 per group). **h**, WT and R403Q mouse concentration response curves were generated by plotting the average total plasma concentration and the average FS response at each time point at doses ranging from 0.25–1.5 mg kg^−1^ (WT, 15 data points; R403Q, 14 data points). Values shown are mean ± s.e.m. Source data

**Extended Data Fig. 9:**
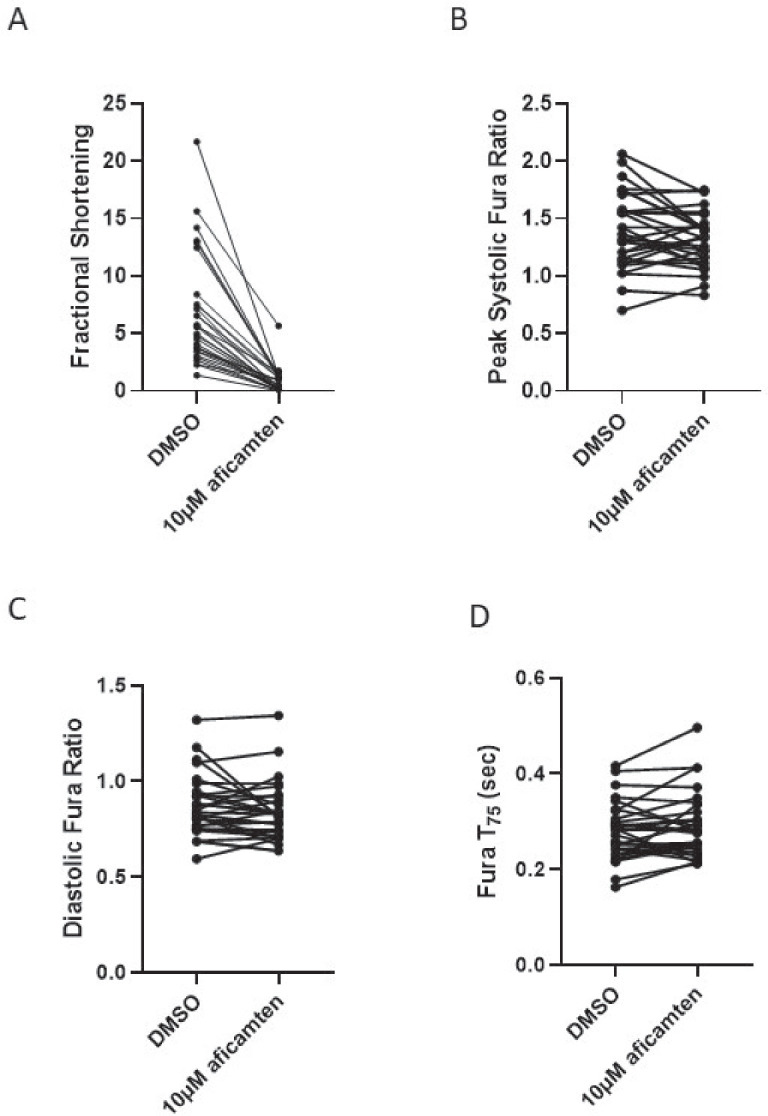
Effect of Aficamten (10 µM) on Simultaneous Fractional Shortening (A) and Calcium Transient Measurements (B–D) in Adult Rat Ventricular Cardiomyocytes. Results from individual cardiomyocytes are shown (n = 30 cells from n = 8 preparations). Tabulated data is shown in Table 3. Source data

Aficamten reduced contractility of normal adult rat ventricular cardiomyocytes without altering calcium transients (Fig. 4a, Extended Data Fig. 9 and Table 3), consistent with a myosin-directed mechanism^28,46^. In separate experiments focused on contractility, aficamten reduced cardiomyocyte fractional shortening with a dose-dependence similar to what was observed for the inhibition of cardiac myosin ATPase activity (Fig. 4b). The acute pharmacodynamic effects of aficamten were examined in healthy Sprague Dawley rats and beagle dogs with echocardiography performed at select time points over a 24–48 h period following a single oral dose. In Sprague Dawley rats, aficamten doses ranging from 0.5-4 mg kg^−1^ produced a dose- and concentration-related reduction in fractional shortening (*P* < 0.05; Fig. 4c,d and Supplementary Table 6). At all dose levels, fractional shortening returned to baseline by 24 h and there were no significant changes in heart rate relative to baseline at any of the dose levels. Of the time points collected, mean total plasma concentrations were highest at 1 h post-dose, and increased in a dose-related manner from 1.2 µM (0.5 mg kg^−1^) to 13.0 µM (4 mg kg^−1^). The total plasma concentration at 10% and 50% reduction of fractional shortening relative to baseline (IC_10_ and IC_50_) was 0.8 µM and 7.9 µM, respectively. Healthy beagle dogs received 0.75, 2 and 3 mg kg^−1^ doses of aficamten and echocardiograms were collected at select time points over a 48-h post-dose period. Similar to the rat studies, aficamten reduced LVEF in a dose- and concentration-related manner 2 h after dosing (Fig. 4e,f and Supplementary Table 7). In healthy beagle dogs, the LVEF IC_10_ and IC_50_ was 0.18 µM and 1.3 µM, respectively. Overall, aficamten reduced cardiac contractility in rats and dogs in a dose and plasma concentration-dependent manner (Fig. 4c–f).

**Table 3:** Effect of aficamten on simultaneous fractional shortening and calcium transient measurements in adult rat ventricular cardiomyocytes

Treatment	*N*	FS (% of basal)	Diastolic Fura ratio	Systolic Fura ratio	Fura *T*_75_ (s)
Basal	30	100	0.87 ± 0.03	1.39 ± 0.06	0.28 ± 0.01
10 μM aficamten	30	12.0 ± 2.0****	0.83 ± 0.03	1.32 ± 0.04	0.29 ± 0.01

Basal reference values are: diastolic cell length = 122.6 ± 2.5 μm, fractional shortening (FS) = 6.73 ± 0.87 μm, contraction velocity = 168.3 ± 30.1 μm s^−1^, relaxation velocity (RV) = 103.9 ± 27.1 μm s^−1^, time to peak = 0.134 ± .0.005 s and time to baseline relaxation (*T*_50_) = 0.245 ± 0.012 s. Aficamten (10 µM) significantly decreases FS (****P < 0.0001) without a significant (*P* < 0.05) change in calcium transient parameters. Data are presented as mean ± s.e.m. (*n* = 30 cells from *n* = 8 preparations).

The ability to reduce pathophysiologic hypercontractility was further tested in mice modeling the first gene variation to be associated with HCM, an arginine-to-glutamine point mutation at codon 403 (R403Q) of exon 13 of the β-myosin heavy-chain gene (*MYH7*)^47^. Cardiac myosin from mice heterozygous for the R403Q mutation has higher actin-activated ATPase and actin gliding velocity in vitro and the mice demonstrate some attributes of HCM, including progressive increases in pathological cardiac hypertrophy and fibrosis in vivo^47,48^. The effect of aficamten was evaluated in R403Q mice at approximately 40–48 weeks of age, where relative to wild-type (WT) mice, R403Q mice had significantly greater septal and posterior wall thickness (*P* < 0.05; Supplementary Table 8) and comparable cardiac fractional shortening (Supplementary Table 9). WT and heterozygous R403Q HCM mice received single oral doses of aficamten ranging from 0.25 to 1.5 mg kg^−1^. In both animal groups, all dose levels of aficamten resulted in a significant, dose-related reduction in fractional shortening 1 h after dosing (*P* < 0.01; Fig. 4g,h and Supplementary Table 9). Fractional shortening returned to pre-dose baseline values at 24 h post-dose at all dose levels in both animal groups and heart rate did not change in any dose groups.

The heart operates within a relatively narrow dynamic range of contractility, and the ability to gradually titrate the pharmacodynamic effect as dose or exposure increases is a critical aspect of a safe and effective therapy. We defined the preclinical pharmacodynamic window of aficamten as the ratio of IC_50_ to IC_10_ for contractility parameters (fractional shortening and LVEF) and found it to be similar across species (rat, 10 (fractional shortening); dog, 7.2 (LVEF); mouse, 7.5 (fractional shortening)) and not affected by the presence of the R403Q mutation (WT, 7.5 versus R403Q, 7.1 (fractional shortening)). Thus, the effect of aficamten to decrease cardiac myosin ATPase activity and cardiomyocyte contractility in vitro translated to a robust effect on contractility in multiple species in vivo with a wide pharmacodynamic window in both healthy rodents and dogs as well as mice harboring a known HCM mutation.

## Discussion

In summary, these data demonstrate the ability of the small molecule aficamten to stabilize cardiac myosin in a weak actin-binding PPS conformation that turns over ATP extremely slowly, reducing the number of active force generators and thus reducing cardiac contractility in vitro and in vivo. Aficamten shares a binding site and general mechanism with the nonselective myosin II inhibitor blebbistatin, but the two differ critically in their selectivity, with aficamten showing selectivity for striated muscle myosins and for cardiac myosin relative to fast skeletal myosin. Both aficamten and mavacamten bind to myosin S1 and inhibit actin-activated phosphate release; however, the phenotype of phosphate release differs. Mavacamten slows phosphate release from S1 approximately fivefold to tenfold (Extended Data Fig. 4, Supplementary Table 4 and ref. ^35^), indicating that heads with mavacamten bound can still bind actin and complete phosphate release. In contrast, our studies indicate that aficamten seems to be able to slow phosphate release to a greater extent based on the combined reduced phosphate release rate, phosphate release amplitude and very slow single ATP turnover rates presented here. The two inhibitors differ in their binding sites based on the structural data presented here describing the aficamten binding site and the inability of mavacamten to compete with blebbistatin for binding to myosin. These results are consistent with structural and molecular dynamics data that have characterized the mavacamten binding site^49^. Allosteric effects of these two specific cardiac myosin inhibitors are thus quite distinct, as reflected by the differences described here for the control of actin-activated P_i_ release.

Studies measuring the turnover of single ATP molecules by myosin using fluorescent (2′-(or-3′)-*O*-(*N*-methylanthraniloyl)) adenosine 5′-triphosphate (mant-ATP)^21,45^ have defined two ATPase states of myosin in cardiac muscle: SRX and the disordered relaxed state (DRX). In the SRX state myosin turns over ATP extremely slowly (~0.003 s^−1^), whereas in the DRX state, myosin turns over ATP tenfold faster (~0.03 s^−1^). SRX is often associated with a folded-back structural state of two-headed myosin molecules known as the interacting-heads motif (IHM), a configuration of myosin heads in which their actin binding is inhibited by both a head–head interaction and head–tail interaction giving rise to an asymmetric configuration of heads folded back onto their own proximal coiled-coil tail^50–54^. Numerous reports have suggested that such a folded-back state has an SRX rate of ATP turnover, whereas the open state with free disordered heads has a DRX rate of ATP turnover^21,44,45,50^.

Previous investigations have suggested that HCM myosin mutations can drive hypercontractility by inducing a decrease in the population of energy-conserving folded-back IHM myosin with a commensurate increase in the population of open-headed myosin^50,55^. It is important to note, however, that SRX is defined as a very low ATP turnover rate that can occur even with single heads of myosin (S1), albeit at a low frequency^19,44^. The folded-back configuration is not essential for myosin heads to exhibit an SRX-like rate of ATP turnover, particularly when drugs are bound. These results provide additional insight into the mechanism by which aficamten can stabilize a slow-cycling, weak actin-binding conformation of myosin. They suggest that aficamten strongly reduces the flexibility of a single myosin head, stabilizing the active site and preventing nucleotide exchange. This mechanism differs from that previously reported for mavacamten^49^, which is less effective in preventing nucleotide exchange in S1, where previous studies demonstrated that mavacamten increased the percentage of slowly exchanging myosin to 30–50% with an exchange rate similar to the typical SRX rate^43,44^. These results are consistent with recent studies comparing inhibition by mavacamten and the blebbistatin analog para-nitroblebbistatin. In addition to stabilizing two different myosin conformations based on X-ray scattering, para-nitroblebbistatin slowed the slow phase of ATP turnover and was postulated to stabilize a small but significant fraction of myosin heads in an ‘ultra-relaxed’ state with >tenfold lower ATP turnover as compared to the typical SRX rate^56^. The properties of aficamten may enable more efficient population of this (or a similar) state.

The results of steady-state ATPase measurements and single mant-ATP turnover rates have frequently been used as a measure of how drugs influence the formation of the IHM conformation^43,44^. Measurements performed in the presence of myosin modulators reveal limitations of that interpretation. Aficamten drastically slows ATP turnover in a single myosin head (S1). It is thus possible for potent small-molecule inhibitors such as aficamten to stabilize myosin and promote slow cycling without necessarily requiring formation of the IHM structural state (which is stabilized by interactions between the two heads of HMM)^52,53^. Additional structural studies will be required to understand how aficamten impacts the ability of the heads to adopt an IHM conformation stabilized on the thick filament, separate from the effects on ATP cycling presented here. The ability of aficamten to stabilize an extremely slow nucleotide-exchanging conformation of myosin likely reduces the number of functionally available motors in the sarcomere in a manner that may be particularly energy-sparing.

In clinical studies in humans, aficamten was well tolerated in healthy adults given single and multiple doses producing reductions in LVEF as dose and exposure increased. The pharmacokinetic half-life of 75–85 h resulted in steady-state within 10–12 days and the effect of aficamten on cardiac function was readily reversible within 24–48 h (ref. ^57^). In a phase 2 clinical trial of patients with oHCM, REDWOOD-HCM (NCT04219826)^58^, aficamten was effective at reducing the LVOT-G pressure gradient and was similarly well tolerated. Recently, results from the phase 3 clinical trial, SEQUOIA-HCM (NCT05186818) were reported. In SEQUOIA-HCM, treatment with aficamten resulted in statistically significant improvements in exercise capacity, New York Heart Association functional class and symptoms with an adverse event profile comparable to placebo^59^.

Although the ramifications of the differences between cardiac myosin inhibitors in patients is still to be determined, the varied molecular underpinnings and mechanisms that lead to oHCM and other diseases of cardiac hypercontractility may ultimately benefit from the development of drug candidates with diverse molecular mechanisms that will be further defined as clinical experience accumulates.

## Methods

### Ethical approval

All rodent experiments were performed at Cytokinetics and conducted in accordance to approved protocols by the Cytokinetics Institutional Animal Care and Use Committee. Beagle dog echocardiography studies were conducted at Charles River Laboratories (formerly MPI Research). Conduct of the study was based on the current International Council for Harmonisation Harmonised Tripartite Guidelines and generally accepted procedures for the testing of pharmaceutical compounds and in accordance with the US Department of Agriculture Animal Welfare Act (9 CFR Parts 1, 2 and 3) and the Guide for the Care and Use of Animal Resources.

### Reagents

All chemicals and reagents were of reagent grade or higher purity. All proteins used in this study were purified by Cytokinetics and demonstrated performance consistent with historical results. Unless stated otherwise, all experiments involving calcium regulation were conducted at a calcium concentration resulting in 75% of maximal calcium-dependent activation (pCa_75_). pCa = −log(free calcium concentration in mol l^−1^).

### Purification of proteins and myofibrils

Myofibrils were prepared from flash-frozen bovine cardiac, bovine masseter (slow skeletal) and rabbit psoas (fast skeletal) tissue as described by Hwee et al.^60^. Bovine cardiac myosin and S1 were prepared as described by Malik et al.^46^. The resulting protein was clarified by ultracentrifugation (Beckman Type 45Ti rotor, 142k *g*_max_, 2.5 h, 4 °C); solid sucrose was added to 10% (*w*/*v*) and the concentration determined by absorbance at 280 nm in 6 M guanidine-HCl using an extinction coefficient of 0.81 cm^2^ mg^−1^. The S1 solution was drop-frozen in liquid nitrogen before storage at −80 °C. Chicken gizzard SMM was purified using variations on Sellers et al.^61^. The resulting protein was clarified by ultracentrifugation (Beckman Type 45Ti rotor, 142k *g*_max_, 2.5 h, 4 °C); solid sucrose was added to 10% (*w*/*v*) and the concentration determined by absorbance at 280 nm in 6 M guanidine-HCl using an extinction coefficient of 0.81 cm^2^ mg^−1^. The S1 solution was drop-frozen in liquid nitrogen before storage at −80 °C. Bovine cardiac actin was prepared from bovine left ventricle acetone powder based on previously described methods^62,63^. The resulting G-actin solution was clarified by centrifugation (Beckman Type 45Ti rotor, 142k *g*_max_, 2 h, 4 °C), drop-frozen in liquid nitrogen and stored at −80 °C. Actin concentrations were determined by absorbance at 290 nm in 6 M guanidine-HCl using an extinction coefficient of 6.3 for a 1% solution, using G-buffer as a blank to control for the ATP present.

### ATPase assays

Steady-state ATPase activity was measured as described in Hwee et al.^60^ and Malik et al.^46^ using a pyruvate kinase and lactate dehydrogenase-coupled enzyme system that regenerates myosin-produced adenosine diphosphate (ADP) into adenosine triphosphate (ATP) by oxidizing the reduced form of nicotinamide adenine dinucleotide (NADH) to the oxidized form of nicotinamide adenine dinucleotide, producing an absorbance change at 340 nm. Myofibril ATPase assays were performed in PM12 buffer (12 mM Pipes, 2 mM MgCl_2_ and 1 mM dithiothreitol (DTT), pH 6.8) supplemented with 60 mM KCl and ATP at approximately 3–10 × *K*_m_ for the particular myofibril system (0.5 mM ATP for fast skeletal and 0.05 mM ATP for slow skeletal and cardiac). Non-myosin ATPase activity was subtracted from cardiac and slow skeletal myofibril assays (where indicated) by subtracting the ATPase activity in the presence of a saturating concentration of the nonselective myosin II inhibitor blebbistatin. Myofibrils were present at approximately 0.25 mg ml^−1^ (fast skeletal) or 1 mg ml^−1^ (slow skeletal, cardiac). Calcium concentrations were controlled using 0.6 mM EGTA and sufficient CaCl_2_ to obtain the desired free calcium concentration (calculated using web resource https://somapp.ucdmc.ucdavis.edu/pharmacology/bers/maxchelator/webmaxc/webmaxcS.htm). Absorbance measurements (340 nm) were carried out at approximately 25 °C using either an Envision plate reader (Perkin-Elmer) using Envision Manager v.1.14 or SpectraMax plate reader (Molecular Devices) using SoftMax Pro v.4.8. Data analysis was performed with GraphPad Prism (GraphPad Software). Dose–response curves were fit with a four-parameter model. EC_50_ indicates the half-maximum effective concentration. In the below equation, Top and Bottom are the top and bottom plateaus in the units of *Y*, and HillSlope describes the steepness of the curve.

1 $$Y={\mathrm{Bottom}}+({\mathrm{Top}}-{\mathrm{Bottom}})/(1+10\wedge (({\mathrm{logEC}}_{50}-x)\times{\mathrm{Hill}}\,{\mathrm{Slope}}))$$

### Binding assays

Binding of (-)-blebbistatin to bovine cardiac myosin S1 was measured in a buffer consisting of 12 mM K-PIPES, pH 6.8, 2 mM MgCl_2_, 1 mM DTT and 2 mM ADP-vanadate. Fluorescence emission spectra were recorded using a PTI QM-6 (λex 426 nm). Compound titrations were performed using a SpectraMax Gemini XS spectrofluorimeter (Molecular Devices, λex 426 nm and λem 575 nm) using SoftMax Pro v.4.8.

### Crystallization and data processing

Crystal of the motor domain (MD) of β-cardiac myosin in the PPS state complexed to aficamten (PPS-MD–aficamten) was obtained by hanging diffusion method at 25 °C from a 1:1 mixture of the S1 fragment (10 mg ml^−1^) with 2 mM Mg.ADP.VO4, 0.5 mM aficamten solubilized in dimethylsulfoxide (DMSO), trypsin (*w*:*w* 1:500) and precipitant containing 23% PEG3350; 0.2 M lithium sulfate and 0.1 M Tris-HCl, pH 7.9. The crystal was transferred in the cryoprotectant solution containing 20% PEG3350; 0.2 M lithium sulfate, 0.1 M Tris-HCl, pH 7.9 and 30% glycerol before flash-freezing in liquid nitrogen. X-ray diffraction data were collected at 100 K on the PX1 beamline (*λ* = 0.97856 Å), at the SOLEIL synchrotron. Diffraction data were processed using the XDS package (v.2020)^64^ and AutoProc (v.1.0.5)^65^. Anisotropic processing was performed with StarAniso (released with v.1.0.5 of AutoProc)^66^. The crystal belongs to the P2_1_ space group with two molecules per asymmetric unit. The cell parameters and data collection statistics are reported in Table 1.

### Structure determination and refinement

Molecular replacement was performed with the MD (residues 33–780) of β-cardiac myosin in the PPS state complexed to omecamtiv mecarbil (Protein Data Bank (PDB) code 5N69) without water and ligand using Phaser (v.2.7.16)^67^ from the Phenix program suite. Manual model building was achieved with Coot (v.0.9.2)^68^. Refinement was performed with BUSTER (v.2.10.2)^69^. The statistics for most favored, allowed and outliers Ramachandran angles are 97.55; 2.30 and 0.14, respectively. The final model has been deposited on the PDB (code 9F6C).

### ATP binding and hydrolysis

ATP binding and hydrolysis were monitored by intrinsic tryptophan fluorescence of bovine cardiac myosin S1 upon binding of various concentrations of ATP using a rapid-mixing stopped-flow instrument (TgK Scientific Ltd. SF-61 DX2) at 25 °C. The excitation wavelength was set to 295 nm to minimize absorption by ATP, and emitted light was collected through a 320 nm long-pass filter. Myosin solutions were clarified by centrifugation (550k *g*_max_, 10 min, 4 °C) after thawing and buffer exchange. Final reaction conditions were 1 μM bovine cardiac myosin S1, 12 mM K-Pipes, pH 6.8, 2 mM MgCl_2_, 1 mM DTT and 2% DMSO. Three to five fluorescence transients were averaged add fit to a single exponential using Kinetic Studio v.5.1.0 (TgK Scientific). In the following equation, *A* is the reaction amplitude, *k* is the rate constant, and *C* is the value of *Y* at infinite time (*x*).

2 $$Y=-A\times \mathrm {exp}(-k\times x) + C$$

### Phosphate release

Actin-activated phosphate release from bovine cardiac myosin S1 was monitored using the 7-diethylamino-3-((((2-maleimidyl)ethyl)amino)carbonyl) coumarin-labeled phosphate binding protein (MDCC–PBP) as in Malik et al.^46^. Experiments were conducted under single turnover conditions where the substrate was limiting. Actin-stimulated phosphate release was measured in a double-mixing experiment where bovine cardiac myosin S1 was mixed with a substoichiometric concentration of ATP, aged for the indicated time (2-30 s) and subsequently mixed with actin and MDCC–PBP (final reaction conditions: 0.5 μM cardiac myosin S1, 0.25 μM ATP, 14 μM actin, 5 μM MDCC–PBP and 2% DMSO). Fluorescence was monitored by excitation at 425 nm, and emitted light was collected through a 455 nm long-pass glass filter. Myosin solutions were clarified by centrifugation (550k *g*_max_, 10 min, 4 °C) after thawing and buffer exchange. Contaminating phosphate was minimized by treatment of all reagents with a purine nucleoside phosphorylase/phosphodeoxyribomutase-coupled enzyme Pi-mop system^70^. F-actin was dialyzed into PM12 buffer followed by addition of 1:10 molar amount of phalloidin before treatment with Dowex resin and a small amount of apyrase (Sigma) to reduce the ATP and ADP contamination present following polymerization. A small amount of residual ATP led to a minor (~10%) slow phase of phosphate release due to additional cycling. Four to six fluorescence transients were averaged add fit to either a single, equation (2) or a double exponential using Kinetic Studio v.5.1.0 (TgK Scientific). In the following equation, *A*_fast_ and *k*_fast_ are the fast reaction amplitude and rate constant. *A*_slow_ and *k*_slow_ are the slow reaction amplitude and rate constant. *C* is the value of *Y* at infinite time (*x*).

3 $$\begin{array}{l}Y=\,-A_{\mathrm{fast}}\times \exp (-k_{{\mathrm{fast}}}\times x)+ -A_{{\mathrm{slow}}}\times \exp (-k_{{\mathrm{slow}}}\times x) + C\end{array}$$

### ADP release

ADP release from myosin S1 and actin-myosin S1 was measured by monitoring changes in the fluorescence of mant-ADP upon displacement from myosin by a high concentration of ATP. A solution containing the cardiac myosin S1–mant-ADP complex or the cardiac myosin S1–mant-ADP–actin complex in the presence or absence of aficamten was rapidly mixed with a concentrated solution of ATP. Myosin solutions were clarified by centrifugation (550k *g*_max_, 10 min, 4 °C) after thawing and buffer exchange. Final reaction conditions were 1 µM bovine cardiac S1, 0.5 µM mant-ADP, 1 mM ATP, 12 mM K-Pipes, pH 6.8, 2 mM MgCl_2_, 1 mM DTT and 2% DMSO. For ADP release from actoS1, bovine cardiac myosin S1, mant-ADP and a fivefold molar excess of bovine cardiac actin were preincubated for ~30 min before rapid mixing with ATP. Fluorescence was monitored by excitation at 363 nm and emitted light was collected through a 400 nm long-pass glass filter. Three to five fluorescence transients were averaged add fit to a single exponential, equation (2) using Kinetic Studio v.5.1.0 (TgK Scientific).

### Single ATP turnover measurements

ATP turnover measurements were performed either in a stopped-flow instrument (TgK Scientific SF-61 DX2) using Kinetic Studio v.5.1.0 or plate-based fluorescence reader (Molecular Devices SpectraMax Gemini XS) using SoftMax Pro v.4.8 (refs. ^50,55^). The ATP turnover rates in the presence of aficamten were slower and suited for a plate-based measurement whereas the ATP turnover experiments in the absence of compound were performed in the stopped-flow system. The experimental buffer comprised 12 mM K-PIPES (pH 6.8), 2 mM MgCl_2_, 1 mM DTT and 60 mM KCl. Both S1 and HMM solutions were centrifuged at 100,000*g* for 15 min at 4 °C to remove any aggregates. Double-mixing experiments were performed in a stopped-flow instrument where myosin was mixed with mant-ATP (2′-(or-3′)-*O*-(*N*-methylanthraniloyl)) adenosine 5′-triphosphate, trisodium salt (Fisher Scientific)) and incubated for 10 s followed by mixing with excess ATP, and then data were collected. The final concentrations of reactants were myosin (0.5 μM), mant-ATP (1 μM), ATP (2 mM) and aficamten (25 μM). The fluorescence signal was collected for 1,000 s using 455 nm cutoff filter with excitation at 374 nm. For plate-based measurements, myosin was mixed with mant-ATP and incubated for 90 s and 300 s for S1 and HMM, respectively, followed by the addition of excess ATP. All the solutions had 25 μM aficamten. The final concentrations of reactants were myosin (0.5 μM), mant-ATP (1 μM), ATP (1 mM) and aficamten (25 μM). Fluorescence was monitored for 3,000 s at 405 nm excitation/455 nm emission. The fluorescence of only 1 μM mant-ATP was collected to confirm insignificant photo bleaching during data collection. At least two sets of experiments were performed for each condition. The fluorescence decrease was fitted to either bi-exponential, equation (3) or single-exponential, equation (2) based on close inspection of the residuals of fitting. A bi-exponential equation was accepted if the single-exponential model was inadequate to fit the data. Fitting fluorescence traces yielded both the relative population and rates of ATP hydrolysis in the SRX and DRX states. All the experiments were performed at room temperature (~23 °C). Myosin solutions were clarified by centrifugation (550k *g*_max_, 10 min, 4 °C) after thawing and buffer exchange.

### In vitro motility

The dynamics of the stabilized actin filament was monitored by TIRF microscopy (Nikon Eclipse Ti2 inverted microscope, ×100 TIRF objectives, sCMOS PRIME 95B camera (Photometrics)). The experiments were controlled using the Metamorph 7 software. Coverslips were sequentially cleaned by sonication with milli-Q water, ethanol and milli-Q water for 10 min, then irradiated for 3 min under a deep UV lamp. Flow chambers were assembled with a coverslip bound to a glass slide with two parallel double-stick tapes. The chamber was incubated with 400 nM bovine cardiac myosin HMM in Fluo F buffer (5 mM Tris-HCl, pH 7.8, 100 mM KCl, 1 mM MgCl_2_, 0.2 mM EGTA, 0.2 mM ATP, 10 mM DTT, 1 mM DABCO and 0.01% NaN_3_) with aficamten at indicated concentrations for 10 min at room temperature. The chamber was rinsed one time with Fluo F buffer and 0.1 % BSA and incubated for 5 min at room temperature. Finally, the chamber was washed with Fluo F buffer. Assays were performed in Fluo F buffer, containing 2 mM constant ATP, supplemented with 0.3% methylcellulose (Sigma) and with 50 nM actin filament (stabilized with phalloidin-Atto488) in presence of aficamten at the indicated concentrations. To maintain a constant concentration of ATP, an ATP-regenerating mix (2 mM ATP, 2 mM MgCl_2_, 10 mM creatine phosphate and 3.5 U ml^−1^ creatine phosphokinase) was added. The video length was 1 min and the time interval between two frames was 1 s. The sliding velocities of actin filaments were analyzed using the ImageJ plugin Kymo Tool Box (https://github.com/fabricecordelieres/IJ-Plugin_KymoToolBox). The accuracy on the displacement of the filaments is of the order of the pixel size (110 nm).

### Myocyte isolation and contractility/calcium measurements

Adult rat cardiomyocytes were isolated using previously described methods^46^. Cells were loaded with 1 µM Fura2-AM for 8 min at room temperature and washed twice in Tyrode’s buffer containing 1.8 mM CaCl_2_. Simultaneous contractility and calcium transient measurements were made at 37 °C using an IonOptix system as described previously^46^. After achieving stable baseline measurements, aficamten was perfused until a stable effect was observed, approximately 5–7 min. Average transients were analyzed using the IonWizard software (IonOptix, v.6.5) to determine changes in diastolic length and fractional shortening. Fractional shortening was calculated as ((resting length − length at peak contraction)/resting length). The percent change in fractional shortening from baseline was calculated as ((post-dose fractional shortening/basal fractional shortening) × 100). The percent reduction in fractional shortening from baseline was calculated as (100 − percent change in fractional shortening from baseline). Results from individual cells were averaged and the s.e.m. or s.d. was calculated.

### Assessment of aficamten in Sprague Dawley Rats

Male Sprague Dawley rats (220–280 g) were obtained from Charles River Laboratories. Rats were housed in a vivarium on a 12-h dark–light cycle. Temperature and humidity were maintained between 68–72 °F and 30–70%, respectively. Single oral doses of vehicle only or aficamten at 0.5, 1, 2 or 4 mg kg^−1^ (in 0.5% hydroxypropylmethylcellulose (HPMC)/0.1% Tween-80 liquid suspension) and cardiac function was assessed 1-day pre-dose, and at 1, 4, 8 and 24 h post-dose. While under isoflurane (1–5%) anesthesia, a 10-MHz probe was placed at the level of the papillary muscles and two-dimensional M-mode images of the LV were captured in long-axis view using a GE Vivid7 ultrasound machine (General Electric). In vivo percent fractional shortening was determined by analysis of the M-mode images using the GE Vivid7 software. Blood samples were obtained via a jugular vein cannula to coincide with echocardiography measurements at 1, 4, 8 and 24 h post-dose for aficamten plasma concentration determination by liquid chromatography with tandem mass spectrometry (LC–MS/MS) analysis.

### Assessment of aficamten in mice bearing the R403Q HCM mutation

Cardiac myosin heavy-chain isoform expression is species dependent. The β-myosin heavy chain is the predominant adult isoform in humans and the α-myosin heavy chain is the predominant adult isoform in mice^71^. To model the human R403Q β-myosin heavy-chain mutation, a mouse model with an R404Q point mutation on the α-myosin heavy chain was generated using CRISPR-Cas9 technology by the Jackson Laboratory. Male WT C57/BL6J and heterozygous R403Q mice were shipped to Cytokinetics at approximately 8 weeks of age. At approximately 40–48 weeks of age, mice were evaluated with aficamten. Mice were housed in a vivarium on a 12-h dark–light cycle. Temperature and humidity were maintained between 68–72 °F and 30–70% respectively.

Over the course of 2 weeks, mice were randomly assigned to one of five oral dose groups ranging from 0.25 to 1.5 mg kg^−1^ (in 0.5% HPMC/0.1% Tween-80) and cardiac fractional shortening was assessed by echocardiography in short-axis view with a Vevo3100 ultrasound system (Fujifilm Visualsonics) before dosing and at 1, 4, 8 and 24 h post-dose. At least 2 days of compound washout occurred before a subsequent dose was evaluated in the same animal. On the final day of evaluation, following each echocardiographic measurement, blood samples were collected for subsequent plasma analysis. For each animal, one non-terminal sample was collected at 1 or 4 h post-dose by retro-orbital bleed and one terminal sample was collected by cardiac puncture at 8 or 24 h post-dose for aficamten plasma concentration determination by LC–MS/MS analysis.

### Assessment of aficamten in beagle dogs

Male beagle dogs (0.5–2.75 years old) received single oral doses of vehicle or aficamten at 0.75, 2 or 3 mg kg^−1^ (in 0.5% HPMC/0.1% Tween-80 liquid suspension). Echocardiographic evaluations were conducted and/or supervised by a board-certified medical sonographer. Cardiac function was assessed in sedated dogs (butorphanol (0.05–0.06 ml) and ketamine (0.05–0.06 ml) at pre-dose, 2, 6, 24 and 48 h post-dose. All dogs received each treatment in a Latin Square study design, with a 7 ± 1-day washout period in between each treatment. Blood samples were obtained via a jugular vein to coincide with echocardiography measurements at 2, 6, 24 and 48 h post-dose for aficamten plasma concentration determination by LC–MS/MS analysis.

### Reporting summary

Further information on research design is available in the Nature Portfolio Reporting Summary linked to this article.

### Supplementary information

Supplementary Information Supplementary Tables 1–9.

Reporting Summary

Supplementary Video 1 In vitro motility assay under control conditions (2% DMSO).

Supplementary Video 2 In vitro motility assay @ 1 µM aficamten.

Supplementary Video 3 In vitro motility assay @ 10 µM aficamten.

Supplementary Video 4 In vitro motility assay @ 100 µM aficamten.

### Source data

Source Data Fig. 1.

Source Data Fig. 3.

Source Data Fig. 4.

Source Data Extended Data Fig. 1.

Source Data Extended Data Fig. 2.

Source Data Extended Data Fig. 4.

Source Data Extended Data Fig. 5.

Source Data Extended Data Fig. 6.

Source Data Extended Data Fig. 7.

Source Data Extended Data Fig. 8.

Source Data Extended Data Fig. 9.

## Data Availability

The atomic model is available in the PDB under accession code 9F6C for the β-MHC–aficamten structure.
