# Supplementary material for: Aficamten is a small-molecule cardiac myosin inhibitor designed to treat hypertrophic cardiomyopathy
Source: Nat Cardiovasc Res. 2024 Jul 23;3(8):1003–16. doi: 10.1038/s44161-024-00505-0 (PMC11358156; doi:10.1038/s44161-024-00505-0)
Supplement: Supplementary file 1 — Supplementary Tables 1–9. [file 44161_2024_505_MOESM1_ESM.pdf]

nature cardiovascular research

Supplementary information

https://doi.org/10.1038/s44161-024-00505-0

Aficamten is a small-molecule cardiac myosin inhibitor designed to treat hypertrophic cardiomyopathy

In the format provided by the authors and unedited

Supplementary Tables

Table S1: Effect of actin concentration on inhibition of steady-state cardiac myosin S1 ATPase activity by myosin inhibitors. Results shown are mean (95% CI) (n=4 technical replicates)

	Aficamten		
Actin Concentration	5 µM	15 µM	45 µM
IC_{50} (µM)	1.14 (1.07 to 1.21)	0.90 (0.85 to 0.92)	0.82 (0.77 to 0.87)
Hill Slope	-1.19 (-1.27 to -1.13)	-1.19 (-1.27 to -1.12)	-1.16 (-1.23 to -1.09)
Maximal Inhibition	92%	98%	99%

	Blebbistatin		
Actin Concentration	5 µM	15 µM	45 µM
IC_{50} (µM)	1.29 (1.23 to 1.34)	1.21 (1.12 to 1.31)	1.32 (1.23 to 1.43)
Hill Slope	-1.07 (-1.11 to -1.03)	-1.04 (-1.10 to -0.97)	-1.07 (-1.15 to -1.01)
Maximal Inhibition	92%	97%	98%

	Mavacamten		
Actin Concentration	5 µM	15 µM	45 µM
IC_{50} (µM)	0.48 (0.46 to 0.50)	0.49 (0.48 to 0.51)	0.62 (0.60 to 0.65)
Hill Slope	-1.04 (-1.08 to -1.00)	-1.04 (-1.07 to -1.00)	-1.03 (-1.07 to -1.00)
Maximal Inhibition	87%	92%	94%

Table S2: Sequence Conservation of the Residues from the Inhibitor Binding Pocket. The equivalent residues in human are displayed.

**B-cardiac myosin**		**SkMyo2**		**MyoII**	**SmMyo2**	
B. taurus (PDB code 7P94)	*H. sapiens* (PDB code 8ACT)	*O. cuniculus* (PDB code 6YSY)	*H. sapiens*	*D. discoidum* (PDB code 1YV3)	*G. gallus* (PDB code 1BR2)	*H.sapiens*
Arg243	Arg243	Arg246	Arg246	Arg238	Arg247	Arg241
Phe244	Phe244	Phe247	Phe247	Phe239	Phe248	Phe242
Gly245	Gly245	Gly248	Gly248	Gly240	Gly249	Gly243
Tyr266	Tyr266	Tyr269	Tyr269	Tyr261	Tyr270	Tyr264
Leu267	Leu267	Leu270	Leu270	Leu262	Leu271	Leu265
Leu268	Leu268	Leu271	Leu271	Leu263	Leu272	Leu266
Glu279	Glu279	Glu272	Glu272	Glu264	Glu273	Glu267
Lys270	Lys270	Lys273	Lys273	Lys265	Lys274	Lys268
Ile462	Ile462	Ile465	Ile465	Ile455	Ile466	Ile460
Ala463	Ala463	Ala466	Ala466	Ser456	Ala467	Ala461
Phe473	Phe473	Leu476	Leu476	Phe466	Phe477	Phe471
Glu474	Glu474	Glu477	Glu477	Glu467	Glu478	Glu472
Cys477	Cys477	Cys480	Cys480	Cys470	Cys481	Cys475
Ile478	Ile478	Ile481	Ile481	Ile471	Ile482	Ile476
Val647	Val647	Val650	Val651	Val630	Val659	Val653
His651	His651	Phe654	Phe655	Tyr634	Tyr663	Tyr657
Asn654	Asn654	Asn657	Asn658	Gln637	Gln666	Gln660
Leu655	Leu655	Leu658	Leu659	Leu638	Leu667	Leu661
Leu658	Leu658	Leu661	Leu662	Leu641	Leu670	Leu674

Table S3: Effect of aficamten on the rate and amplitude of actin-activated phosphate release as a function of age time. Results are mean ± SD (n=5 technical replicates)

	Vehicle				Aficamten (40 µM)			
Age Time (sec)	k_{fast} (s^{-1})	k_{slow} (s^{-1})	A_{fast} (µM P_i)	A_{slow} (µM P_i)	k_{fast} (s^{-1})	k_{slow} (s^{-1})	A_{fast} (µM P_i)	A_{slow} (µM P_i)
2	1.69 ± 0.065	0.13 ± 0.019	0.49 ± 0.005	0.06 ± 0.008	1.63 ± 0.058	0.19 ± 0.023	0.45 ± 0.011	0.052 ± 0.004
10	1.69 ± 0.085	0.21 ± 0.009	0.39 ± 0.004	0.06 ± 0.001	1.51 ± 0.054	0.21 ± 0.019	0.27 ± 0.009	0.074 ± 0.01
20	1.54 ± 0.078	0.23 ± 0.027	0.32 ± 0.004	0.09 ± 0.02	1.13 ± 0.024	0.21 ± 0.022	0.18 ± 0.005	0.086 ± 0.013
30	1.39 ± 0.034	0.27 ± 0.017	0.29 ± 0.015	0.13 ± 0.013	0.89 ± 0.028	0.18 ± 0.024	0.15 ± 0.025	0.086 ± 0.013

Table S4: Effect of blebbistatin and mavacamten on the rate and amplitude of actin-activated phosphate release as a function of age time. Results are mean ± SD (n=5 technical replicates)

	Age Time (sec)							
	2				30			
	k_{fast} (s^{-1})	k_{slow} (s^{-1})	A_{fast} (µM P_i)	A_{slow} (µM P_i)	k_{fast} (s^{-1})	k_{slow} (s^{-1})	A_{fast} (µM P_i)	A_{slow} (µM P_i)
Vehicle	1.59 ± 0.043	0.17 ± 0.012	0.54 ± 0.005	0.054 ± 0.005	1.35 ± 0.034	0.27 ± 0.009	0.30 ± 0.009	0.17 ± 0.036
Blebbistatin (40 µM)	1.58 ± 0.057	0.13 ± 0.008	0.31 ± 0.008	0.04 ± 0.001	0.61 ± 0.053	0.056 ± 0.009	0.14 ± 0.021	0.084 ± 0.005
Mavacamten (40 µM)	0.18 ± 0.004		0.62 ± 0.008		0.21 ± 0.004		0.69 ± 0.023	

Table S5: Effect of Aficamten on the Single Nucleotide Turnover Rate of Cardiac Myosin HMM

**Treatment**	**N**	**% Slow Turnover**	**k_{fast} (s^{-1})**	**k_{slow} (s^{-1})**
Vehicle	8	12.3 ± 1.8	0.020 ± 0.0017	0.0035 ± 0.00057
25 μM aficamten	19	100#*		0.00038 ± 0.00014*

Data are presented as mean ± SD. #Traces for reactions containing aficamten were equally well-fit by single and double exponential equations. *p<0.0001 vs. vehicle calculated using a two-tailed unpaired t-test.

Table S6: Echocardiographic Measurements of Fractional Shortening Prior To (baseline) and 1, 4, 8, and 24 Hours After Single Oral Doses of Aficamten in Healthy Rats. (Tabular Data for Fig. 4C)

**Dose (mg/kg)**	**Rat Fractional Shortening (%)**				
	**Pre-dose Baseline**	**1 hr**	**4 hr**	**8 hr**	**24 hr**
Vehicle	50.7 ± 1.1	47.9 ± 1.3	47.5 ± 1.8	50.8 ± 1.8	50.4 ± 1.5
0.5	49.0 ± 1.0	39.0 ± 1.6<br>p=0.048	43.8 ± 4.6	46.8 ± 1.3	51.3 ± 0.6
1	49.3 ± 1.8	31.3 ± 2.5<br>p=0.012	40.3 ± 2.9	44.5 ± 3.4	52.3 ± 1.7
2	51.3 ± 1.6	29.2 ± 1.8<br>p=0.001	35.3 ± 2.0<br>p=0.001	--	49.3 ± 1.2
4	49.1 ± 1.2	15.0 ± 3.7<br>p=0.004	28.8 ± 3.5<br>p=0.01	36.5 ± 1.6<br>p=0.008	52.3 ± 1.9

Values are expressed mean ± SEM. P-values are comparisons to pre-dose baseline values within each dose and timepoint by multiple comparison mixed-effects analysis.

Table S7 Echocardiographic Measurements of Ejection Fraction Prior To (Baseline) and 2, 6, 24, and 48 Hours After Single Oral Doses of Aficamten in Beagle Dogs. (Tabular Data for Fig. 4E)

Dose (mg/kg)	Dog Ejection Fraction (%)				
	**Pre-dose Baseline**	**2 hr**	**6 hr**	**24 hr**	**48 hr**
Vehicle	74.1 ± 2.2	74.5 ± 2.5	75.7 ± 0.9	74 ± 1.7	73.5 ± 1.4
0.75	73.4 ± 1.6	62.5 ± 2.5 p=0.006	61.9 ± 2.9 p=0.009	68.1 ± 1.8	70.1 ± 2.1
2	73.3 ± 2.7	45 ± 3.0 p=0.0002	52.5 ± 1.6 p=0.002	55.5 ± 2.4 p=0.004	62.5 ± 2.3 p=0.03
3	76.0 ± 2.3	36.8 ± 2.4 p<0.0001	40.3 ± 4.1 p=0.001	51.9 ± 3.0 p=0.0004	64.4 ± 3.3 p=0.048

Values are expressed mean ± SEM. p-values are comparisons to pre-dose baseline values within each dose and timepoint by multiple comparison mixed-effects analysis.

Table S8: Wall Thickness in WT and R403Q Mice Prior to Dosing

Mice Type	Septal Wall Thickness (mm)	Posterior Wall Thickness (mm)
WT (n=10)	0.91 ± 0.02 mm	0.81 ± 0.03 mm
R403Q (n=9)	1.18 ± 0.05 mm<br>p=0.0001	1.04 ± 0.03 mm<br>p<0.0001

Values shown are mean ± SEM. P-values vs. WT by unpaired t-test.

Table S9: Echocardiographic Measurements of Fractional Shortening Prior To (Baseline) and 1, 4, 8, and 24 Hours After Single Oral Doses of Aficamten in WT and R403Q Mice. (Tabular Data for Fig. 4G)

**WT Mouse Fractional Shortening (%)**					
**Dose (mg/kg)**	**Baseline**	**1 hr**	**4 hr**	**8 hr**	**24 hr**
0.25	54.9 ± 0.9	46.6 ± 2.2<br>p=0.002	51.9 ± 1.8	51.2 ± 1.3	52.7 ± 1.3
0.5	56.0 ± 1.7	37.0 ± 1.0<br>p<0.0001	43.1 ± 0.8<br>p= 0.0003	47.7 ± 4.1<br>p= 0.03	54.9 ± 1.2
1	55.9 ± 1.1	27.5 ± 0.8<br>p<0.0001	36.2 ± 1.7<br>p<0.0001	43.1 ± 1.1<br>p<0.0001	54.9 ± 2.2
1.25	54.8 ± 1.5	26.0 ± 1.4<br>p<0.0001	35.7 ± 0.7<br>p<0.0001	42.5 ± 2.3<br>p=0.0007	54.9 ± 2.2
1.5	57.9 ± 0.7	14.5 ± 1.5<br>p<0.0001	25.0 ± 2.7<br>p<0.0001	32.0 ± 2.2<br>p<0.0001	54.6 ± 0

Values are expressed mean ± SEM. p-values are comparisons to baseline values within each dose and timepoint by 2-way ANOVA

**R403Q Mouse Fractional Shortening (%)**					
**Dose (mg/kg)**	**Baseline**	**1 hr**	**4 hr**	**8 hr**	**24 hr**
0.25	55.5 ± 2.4	43.9 ± 1.5<br>p<0.0001	49.9 ± 2.0	54.0 ± 1.5	56.4 ± 2.2
0.5	50.8 ± 3.1	34.6 ± 5.0<br>p<0.0001	39.1 ± 3.5<br>p=0.0013	40.7 ± 1.5<br>p=0.007	53.6 ± 0.7
1	57.8 ± 2.1	27.3 ± 2.2<br>p<0.0001	35.2 ± 3.3<br>p<0.0001	43.2 ± 2.7<br>p<0.0001	54.4 ± 3.1
1.25	54.7 ± 2.8	28.3 ± 2.6<br>p<0.0001	37.8 ± 1.1<br>p<0.0001	43.9 ± 1.2<br>p=0.003	53.1 ± 1.4
1.5	60.1 ± 1.5	13.7 ± 0.6<br>p<0.0001	28.6 ± 0.9<br>p<0.0001	34.3 ± 0.5<br>p<0.0001	57.4 ± 0

Values are expressed mean ± SEM. p-values are comparisons to baseline values within each dose and timepoint by 2-way ANOVA
