# Supplementary material for: Aficamten is a small-molecule cardiac myosin inhibitor designed to treat hypertrophic cardiomyopathy
Source: Nat Cardiovasc Res. 2024 Jul 23;3(8):1003–16. doi: 10.1038/s44161-024-00505-0 (PMC11358156; doi:10.1038/s44161-024-00505-0)
Supplement: Supplementary file 2 — Reporting Summary [file 44161_2024_505_MOESM2_ESM.pdf]

nature portfolio

Corresponding author(s): James J. Hartman

Last updated by author(s): Jun 5, 2024

Reporting Summary

Nature Portfolio wishes to improve the reproducibility of the work that we publish. This form provides structure for consistency and transparency in reporting. For further information on Nature Portfolio policies, see our Editorial Policies and the Editorial Policy Checklist.

Statistics

For all statistical analyses, confirm that the following items are present in the figure legend, table legend, main text, or Methods section.

n/a	Confirmed	
☐	☒	The exact sample size (*n*) for each experimental group/condition, given as a discrete number and unit of measurement
☐	☒	A statement on whether measurements were taken from distinct samples or whether the same sample was measured repeatedly
☐	☒	The statistical test(s) used AND whether they are one- or two-sided<br>*Only common tests should be described solely by name; describe more complex techniques in the Methods section.*
☐	☒	A description of all covariates tested
☐	☒	A description of any assumptions or corrections, such as tests of normality and adjustment for multiple comparisons
☐	☒	A full description of the statistical parameters including central tendency (e.g. means) or other basic estimates (e.g. regression coefficient) AND variation (e.g. standard deviation) or associated estimates of uncertainty (e.g. confidence intervals)
☐	☒	For null hypothesis testing, the test statistic (e.g. *F*, *t*, *r*) with confidence intervals, effect sizes, degrees of freedom and *P* value noted<br>*Give P values as exact values whenever suitable.*
☒	☐	For Bayesian analysis, information on the choice of priors and Markov chain Monte Carlo settings
☒	☐	For hierarchical and complex designs, identification of the appropriate level for tests and full reporting of outcomes
☒	☐	Estimates of effect sizes (e.g. Cohen's *d*, Pearson's *r*), indicating how they were calculated

Our web collection on statistics for biologists contains articles on many of the points above.

Software and code

Policy information about availability of computer code

Data collection	Data collection software is described in materials and methods as appropriate.
Data analysis	Data analysis software is described in materials and methods as appropriate. The primary tool for analysis of non-structural data was Graphpad Prism v8. Plate-based measurements were carried out using either an Envision plate reader controlled by Envision Manager v.1.14 (Perkin Elmer) or SpectraMax plate reader controlled by SoftMax Pro v.4.8 (Molecular Devices). Pre-steady-state measurements were carried out using a SF61 DX2 stopped-flow instrument controlled by Kinetic Studio v.5.1.0 (TgK Scientific Ltd). In vitro motility assay movies were collected using Metamorph 7, and the resulting images were analyzed using the Image J plugin Kymo Tool Box v1.0.1. X-ray diffraction data were processed using the XDS package (v. 2020) and AutoProc (v. 1.0.5). Anisotropic processing was performed with StarAniso (released with v. 1.0.5. of AutoProc). Molecular replacement was performed with Phaser (v. 2.7.16) from the Phenix program suite. Manual model building was achieved with Coot (v. 0.9.2). Refinement was performed with Buster (v 2.10.2).

For manuscripts utilizing custom algorithms or software that are central to the research but not yet described in published literature, software must be made available to editors and reviewers. We strongly encourage code deposition in a community repository (e.g. GitHub). See the Nature Portfolio guidelines for submitting code & software for further information.

Data

Policy information about availability of data

All manuscripts must include a data availability statement. This statement should provide the following information, where applicable:

- Accession codes, unique identifiers, or web links for publicly available datasets
- A description of any restrictions on data availability
- For clinical datasets or third party data, please ensure that the statement adheres to our policy

The atomic model is available in the PDB, www.pdb.org, under accession numbers PDB 9F6C for the B-MHC-aficamten structure.

Research involving human participants, their data, or biological material

Policy information about studies with human participants or human data. See also policy information about sex, gender (identity/presentation), and sexual orientation and race, ethnicity and racism.

Reporting on sex and gender	N/A
Reporting on race, ethnicity, or other socially relevant groupings	N/A
Population characteristics	N/A
Recruitment	N/A
Ethics oversight	N/A

Note that full information on the approval of the study protocol must also be provided in the manuscript.

Field-specific reporting

Please select the one below that is the best fit for your research. If you are not sure, read the appropriate sections before making your selection.

☒ Life sciences ☐ Behavioural & social sciences ☐ Ecological, evolutionary & environmental sciences

For a reference copy of the document with all sections, see nature.com/documents/nr-reporting-summary-flat.pdf

Life sciences study design

All studies must disclose on these points even when the disclosure is negative.

Sample size	For in vitro studies, sample sizes were based on historical signal/noise information and previous studies with other cardiac myosin inhibitors. For in vivo studies, sample sizes with aficamten were selected based on previous screening data with other cardiac myosin inhibitors.
Data exclusions	For in vitro studies, the only data excluded were in cases of technical failure or failure to meet established quality parameters. For in vivo studies, no data was excluded.
Replication	Within animal species, no reproducibility studies were conducted. The reproducibility of an effect by aficamten on cardiac function was demonstrated across multiple species, including mice, rats, and dogs.
Randomization	For rodent studies, animals were randomized by baseline fractional shortening measurements. Randomization of treatment was not performed in beagle dogs, as a latin square study design was employed and all dogs received vehicle treatment and all three dose levels over the course of the study.
Blinding	For rodent echocardiography studies, experimenters were blinded to dosing and/or image analysis. For biochemical studies, blinding was not possible as experiments were setup and executed by individuals, although different experiments were carried out by different individuals based on technical expertise.

Reporting for specific materials, systems and methods

We require information from authors about some types of materials, experimental systems and methods used in many studies. Here, indicate whether each material, system or method listed is relevant to your study. If you are not sure if a list item applies to your research, read the appropriate section before selecting a response.

Materials & experimental systems

n/a	Involved in the study
☒	☐ Antibodies
☒	☐ Eukaryotic cell lines
☒	☐ Palaeontology and archaeology
☐	☒ Animals and other organisms
☒	☐ Clinical data
☒	☐ Dual use research of concern
☒	☐ Plants

Methods

n/a	Involved in the study
☒	☐ ChIP-seq
☒	☐ Flow cytometry
☒	☐ MRI-based neuroimaging

Animals and other research organisms

Policy information about studies involving animals; ARRIVE guidelines recommended for reporting animal research, and Sex and Gender in Research

Laboratory animals	Mouse: Male and Female R403Q HCM model and noncarrier controls (WT) predominantly on a C57/BL6J background strain; age range: ~40-48 weeks. Mice were housed in a vivarium on a 12-hour dark/light cycle. Temperature and humidity were maintained between 68-72°F and 30-70% respectively.<br>Rat: Male Sprague Dawley Rats; weight range: 200-250 grams. Rats were housed in a vivarium on a 12-hour dark/light cycle. Temperature and humidity were maintained between 68-72°F and 30-70% respectively.<br>Dog: Male Beagle Dogs; age range: 0.5-2.75 years old
Wild animals	These studies did not involve any wild animals.
Reporting on sex	Findings do not apply only to one sex. Studies in male and female mice did not show differences in the pharmacological response to aficamten.
Field-collected samples	These studies did not involve field-collected samples.
Ethics oversight	All animal rodent experiments were performed at Cytokinetics (South San Francisco, CA, USA) and conducted in accordance to approved protocols by the Cytokinetics Institutional Animal Care and Use Committee (IACUC). Beagle dog echocardiography studies were conducted at Charles River Laboratories (formerly MPI Research, Mattawan, Michigan).Conduct of the study was based on the current International Council for Harmonisation (ICH) Harmonised Tripartite Guidelines and generally accepted procedures for the testing of pharmaceutical compounds and in accordance with the US Department of Agriculture (USDA) Animal Welfare Act (9 CFR Parts 1, 2, and 3) and the Guide for the Care and Use of Animal Resources.

Note that full information on the approval of the study protocol must also be provided in the manuscript.

Plants

Seed stocks	*Report on the source of all seed stocks or other plant material used. If applicable, state the seed stock centre and catalogue number. If plant specimens were collected from the field, describe the collection location, date and sampling procedures.*
Novel plant genotypes	*Describe the methods by which all novel plant genotypes were produced. This includes those generated by transgenic approaches, gene editing, chemical/radiation-based mutagenesis and hybridization. For transgenic lines, describe the transformation method, the number of independent lines analyzed and the generation upon which experiments were performed. For gene-edited lines, describe the editor used, the endogenous sequence targeted for editing, the targeting guide RNA sequence (if applicable) and how the editor was applied.*
Authentication	*Describe any authentication procedures for each seed stock used or novel genotype generated. Describe any experiments used to assess the effect of a mutation and, where applicable, how potential secondary effects (e.g. second site T-DNA insertions, mosiacism, off-target gene editing) were examined.*
